# Using a Sequential Regimen to Eliminate Bacteria at Sublethal Antibiotic Dosages

**DOI:** 10.1371/journal.pbio.1002104

**Published:** 2015-04-08

**Authors:** Ayari Fuentes-Hernandez, Jessica Plucain, Fabio Gori, Rafael Pena-Miller, Carlos Reding, Gunther Jansen, Hinrich Schulenburg, Ivana Gudelj, Robert Beardmore

**Affiliations:** 1 Centro de Ciencias Genómicas, Universidad Nacional Autónoma de México, Cuernavaca, México; 2 Biosciences, Geoffrey Pope Building, University of Exeter, Exeter, United Kingdom; 3 Evolutionary Ecology and Genetics, Christian-Albrechts-Universität zu Kiel, Kiel, Germany; The Pennsylvania State University, UNITED STATES

## Abstract

We need to find ways of enhancing the potency of existing antibiotics, and, with this in mind, we begin with an unusual question: how low can antibiotic dosages be and yet bacterial clearance still be observed? Seeking to optimise the simultaneous use of two antibiotics, we use the minimal dose at which clearance is observed in an in vitro experimental model of antibiotic treatment as a criterion to distinguish the best and worst treatments of a bacterium, *Escherichia coli*. Our aim is to compare a combination treatment consisting of two synergistic antibiotics to so-called sequential treatments in which the choice of antibiotic to administer can change with each round of treatment. Using mathematical predictions validated by the *E*. *coli* treatment model, we show that clearance of the bacterium can be achieved using sequential treatments at antibiotic dosages so low that the equivalent two-drug combination treatments are ineffective. Seeking to treat the bacterium in testing circumstances, we purposefully study an *E*. *coli* strain that has a multidrug pump encoded in its chromosome that effluxes both antibiotics. Genomic amplifications that increase the number of pumps expressed per cell can cause the failure of high-dose combination treatments, yet, as we show, sequentially treated populations can still collapse. However, dual resistance due to the pump means that the antibiotics must be carefully deployed and not all sublethal sequential treatments succeed. A screen of 136 96-h-long sequential treatments determined five of these that could clear the bacterium at sublethal dosages in all replicate populations, even though none had done so by 24 h. These successes can be attributed to a collateral sensitivity whereby cross-resistance due to the duplicated pump proves insufficient to stop a reduction in *E*. *coli* growth rate following drug exchanges, a reduction that proves large enough for appropriately chosen drug switches to clear the bacterium.

## Abstract 2

A laboratory treatment model shows that the sequentially alternating use of two antibiotics can be optimized to outperform combination treatments at the equivalent dose.

## Author Summary

So-called “cocktail” treatments are often proposed as a way of enhancing the potency of antibiotics, based on the idea that multiple drugs can synergise when used together as part of a single combined therapy. We investigated whether any other multidrug deployment strategies are as effective as—or perhaps even better than—synergistic antibiotic combinations at reducing bacterial densities. “Collateral sensitivities” between antibiotics are frequently observed; this is when measures taken by a bacterium to counter the presence of one antibiotic sensitise it to the subsequent use of another. Our approach was to see if we could exploit these sensitivities by first deploying one drug, then removing it and instead deploying another, and then repeating this process. This is not an entirely new idea, and there is a precedence for this form of treatment that has been trialled in the clinic for *Helicobacter pylori* infection. The idea we pursued here is an extension of “sequential treatment”; we investigated whether with two antibiotics and *n* rounds of treatment, if we search within the set of all possible *2* ^*n*^ “sequential treatments”—including the two single-drug monotherapies—there might be treatments within that set that are more effective than the equivalent two-drug cocktail. Using a simple in vitro treatment model, we show that some sequential-in-time antibiotic treatments are successful under conditions that cause the failure of the cocktail treatment when implemented at the equivalent dosage.

## Introduction

Bacteria have a remarkable capacity to adapt and evolve. It is probably unsurprising in retrospect that resistance has developed to every antibiotic in clinical use [1], with the genes responsible disseminated globally [2,3]. Antibiotic resistance, therefore, has the potential to become a very grave problem. Bacteria evolve so rapidly, in fact, that whole-genome sequencing studies have been able to elucidate dozens of de novo drug-resistance mutations occurring at high frequency within a clinical patient’s infection during a 12-wk treatment [4]. Given this, the following seems an important question: what ways of combining antibiotics might be used to combat infection even when the bacterial species in question exhibits rapid decreases in drug susceptibility during treatment? Or, to put it differently, how can we enlarge the “optimisation space” of antibiotic combinations and search within those for novel, effective treatments?

One possibility may lie with so-called sequential treatments. They have been the subject of several recent laboratory studies [5–7] and clinical trials [8,9] in which the idea is to alternate the use of different antibiotic classes through time. Thus, if, for example, two antibiotics are available and *n* rounds of treatment are to be given, then there are 2^*n*^ different ways of administering the drugs. Our hypothesis states that this exponentially large optimisation space can contain more effective treatments than the equivalent two-drug combination treatment when the same dosages of each antibiotic are applied.

We demonstrate the veracity of this claim in one particular in vitro laboratory model that mimics something of the gravity of the situation we now face by using a bacterium that possesses a scalable drug efflux mechanism that quickly reduces the efficacy of the antibiotics at our disposal. Despite this mechanism, we show that sequential treatments can clear the bacterium when the equivalent combination treatment fails to, provided, that is, that the drugs are deployed in a suitably optimised, sequential manner.

To demonstrate this, we use the following laboratory system. *Escherichia coli* K12 (AG100) is targeted with two antibiotics, erythromycin (a macrolide, ERY) and doxycycline (a tetracycline, DOX), that bind to different ribosomal RNA subunits, thereby inhibiting translation. While this is a nonclinical drug pairing, the commercial drug Synercid (comprising quinupristin and dalfopristin) also targets ribosomal RNA combinatorially [10]. Moreover, some clinical combinations have ambiguous pharmacological interactions that can appear antagonistic in vitro [11,12], whereas the ERY—DOX pairing has an established synergy [13,14].

Before continuing, we need to declare a standard notational device that we will use throughout. It defines how antibiotic efficacy is measured, independently of the drug under study. Thus, IC_*x*_ will denote the antibiotic concentration that reduces the density of the ancestral bacterial strain (AG100), rather than (for example) any other fitness measure, exponential growth rate, or area under a growth curve, by a factor *x*% relative to that produced without antibiotic in any single period of bacterial growth.

Now, *E*. *coli* is known to decrease susceptibility to ERY and DOX by amplifying a genomic region that contains the operon *acrRAB* because a multidrug pump is formed from the products of *acrRAB* and *tolC* [13,15]. Selection for amplification mutations occurs even when the drugs are combined at high concentrations whereupon pump duplications and triplications are observed [16]. The triplications permit bacteria subjected to 5 d of combination treatment at twice IC_95_ dosages, and thus at very low population densities, to eventually restore their growth rates and population densities to almost untreated levels [16].

In these circumstances, the successful clearance of *E*. *coli* using sublethal dosages of ERY and DOX appears implausible. Low-dose monotherapies are unlikely to work [17], and combining the antibiotics into a synergistic IC_50_ cocktail (that achieves IC_90_ overall because of the synergy) is known to be futile because of resistance increases provided by the pump duplications [13]. We therefore turn to sequential treatments, an approach that has been used to treat cancers [18–20] and some clinical infections [9]. These might also appear predestined to fail; after all, cross drug collateral sensitivities are believed to be the basis of successful sequential treatments [7], whereas our model system, by contrast, has a scalable multidrug pump at its disposal.

Nevertheless, to evaluate the impact of extended antibiotic treatments, we propagated populations of *E*. *coli* in 96-well microtitre plates containing liquid medium supplemented with antibiotics based on 12-h cycles, aka seasons, of growth. Thus, two drug treatments per day were administered. At the end of each season, 1% of the spent liquid media, containing biomass, was transferred to a plate containing fresh medium and antibiotics, where growth could resume. The media was supplemented with enough glucose that this protocol would not clear the bacterium in the absence of drug but would instead establish a near-constant, season-by-season total observed population density of about 10^8^ cells per ml in stationary phase (as can be discerned from Fig S1 and Fig S7 in S1 Text). Given this model, we sought antibiotic treatments capable of clearing the bacterium.

## Results

### Low-Dose (IC^50^) and Mid-Dose (IC^70^) Sequential Drug Screens

By the term sequential treatment, we mean the following protocol: one of the two drugs is used in season 1, and, whether ERY or DOX, it may be re-used in season 2, or, alternatively, the other drug may be deployed instead. This process then continues each season until treatment ends. For a treatment of eight seasons, there are 2^8^–2 = 254 possible sequential protocols (minus the two monotherapies). However, seeking to understand whether drug switches per se reduce population growth, only balanced sequential treatments that use four seasons of both drugs were trialled (Fig S6 in S1 Text, section 1). Seeking evidence of successful low-dose treatments, we first treated *E*. *coli* with ERY and DOX for eight seasons at dosages corresponding to the IC_50_ of each drug, implementing the following treatments: two monotherapies, one 50/50 combination using a half dose of both drugs (achieving approximately IC_90_, Fig S3 and Fig S4 in S1 Text, section 1) in addition to 70 sequential treatments (three replicates each). An analogous screen of sequential treatments was then implemented at IC_70_ dosages (but only 66 of these sequential treatments were implemented).

**Fig 1:**
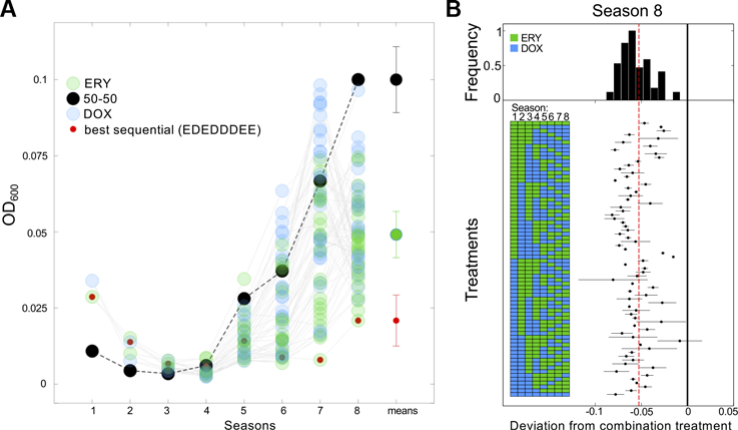
At IC_50_ dosages, population recovery is fastest for the 50/50 combination treatment and slowest for a sequential treatment. (A) Mean densities are shown at the end of each season for all sequential treatments at IC_50_ (as blue and green dots) and for the 50/50 combination of both drugs (black dotted line). The treatment maximising inhibition in season 1 (at 12 h) is the 50/50 combination treatment, because of the synergy. However, by season 8 (at 96 h), all sequential treatments produce lower mean densities than the 50/50 treatment, out of which the lowest density obtained from all the treatments tested is indicated by red circles. Also shown are mean final densities (see *x*-label “means”) of the 50/50 treatment (black circle), the best sequential treatment (red circle), and of all sequential treatments (green circle ± SE, three replicates per treatment). (B) A forest plot showing densities obtained using different sequential treatments at 96 h relative to the 50/50 treatment (drug orders are illustrated by the blue and green boxes on the left). The vertical black line represents the mean density for the 50/50 combination, the vertical dashed line is the mean of all the sequential treatments, and the dots mark the deviation in density produced from the 50/50 combination treatment (± SE, *n* = 3). Like (A), this shows that the combination treatment performs at the poorest extreme of the distribution of all sequential treatments measured in terms of how bacterial growth is suppressed by 96 h. There is no evidence of bacterial clearance in any treatment. (S1 Data contains the data used in this figure.)

Fig. 1 summarises the IC_50_ data. In Fig. 1A, the 50/50 combination treatment achieves greater single-season inhibition than each monotherapy, as expected from prior reports of synergy (*p*<10^-7^, test as indicated in Fig S3 in S1 Text). However, by 36 h the combination therapy no longer produces the lowest bacterial densities, and by 96 h it produces high final densities (Fig. 1A and Fig. 1B), higher than the mean of the family of sequential treatments (*p*<10^-8^,*F*(1,69)≈47.1, one-way ANOVA). Although a sequential treatment has the lowest final density of all those trialled (Fig. 1A), no IC_50_ treatment provided any evidence of eliminating the bacteria by 96 h.

**Fig 2:**
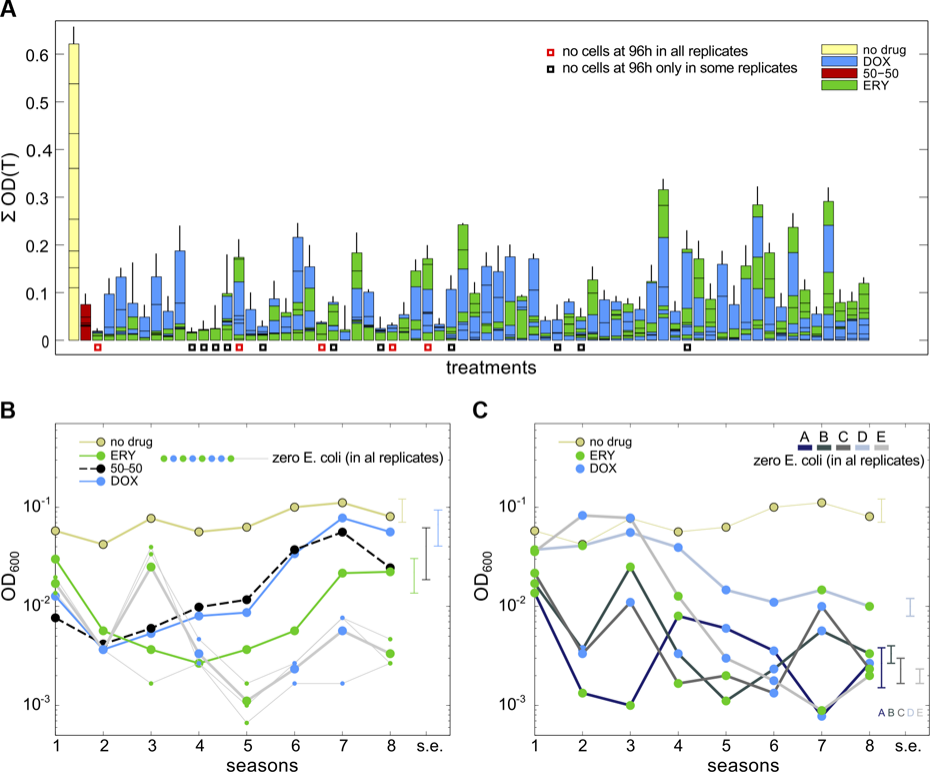
Some examples of successful sequential treatments at IC_70_ dosages. (A) This Manhattan plot at I*C*_70_ shows the mean total optical densities observed during eight seasons of treatment (Σ*0D*(*T*) on the vertical axis, vertical lines are SE, and *n* = 3). Note the 16 treatments marked with a red or black square: they had among the lowest final densities of the treatments trialled. After this, the 16 treatments were replicated; a red square shows that a zero cell density was observed in all three initial and subsequent replicates of that treatment, and black squares show a zero population density was observed in some, but not all, replicates. (B) The no-drug, ERY, and DOX monotherapies and the 50/50 combination treatment all produce recovering mean population densities at IC_70_ doses. These four unsuccessful treatments are shown next to the optical density dynamics of three replicates of a successful “red square” treatment from (A) (treatment C in panel C). The three replicates (shown as grey lines with blue [DOX] and green [ERY] circles) indicate parallel dynamics and fluctuating decay towards zero (bars are SE of optical density at 96 h, *n* = 3). (C) Season-by-season mean densities of all the successful (red square) treatments from (A); note how two achieve high densities early during treatment. Fig S15 in S1 Text, section 3, shows colony-forming units for replicates of these treatments. (S1 Data contains the data used in this figure.)

After increasing dosages to their IC_70_ values, the following evidence of bacterial clearance by 96 h was observed. Sixteen sequential treatments that produced some of the lowest population densities after 96 h of treatment (treatments marked with boxes in Fig. 2A) were examined, and, using spot tests, we could isolate no live cells for five of these treatments in all three replicates. The 11 remaining treatments lead to a zero cell count in some replicates but not in all (Fig S15 in S1 Text, section 3). We then replicated all 16 treatments an additional three times, and the five previously successful treatments again produced a zero cell count by 96 h, although the remaining 11 treatments showed substantial between-replicate variability in their population dynamics (Fig S15). By contrast, Fig. 2B shows that the 50/50 combination treatment (with a greater inhibition than IC_70_ due to the synergy) and both monotherapies yielded recovering (i.e., increasing) mean population densities beyond 48 h at these dosages. (In addition, we recall that twice IC_95_ combinations of these drugs can fail in this treatment model too [16].) However, these observations serve to illustrate that appropriately optimised, sequential therapies at IC_70_ can clear a bacterium even when synergistic combination treatments with greater one-season inhibition do not.

**Fig 3:**
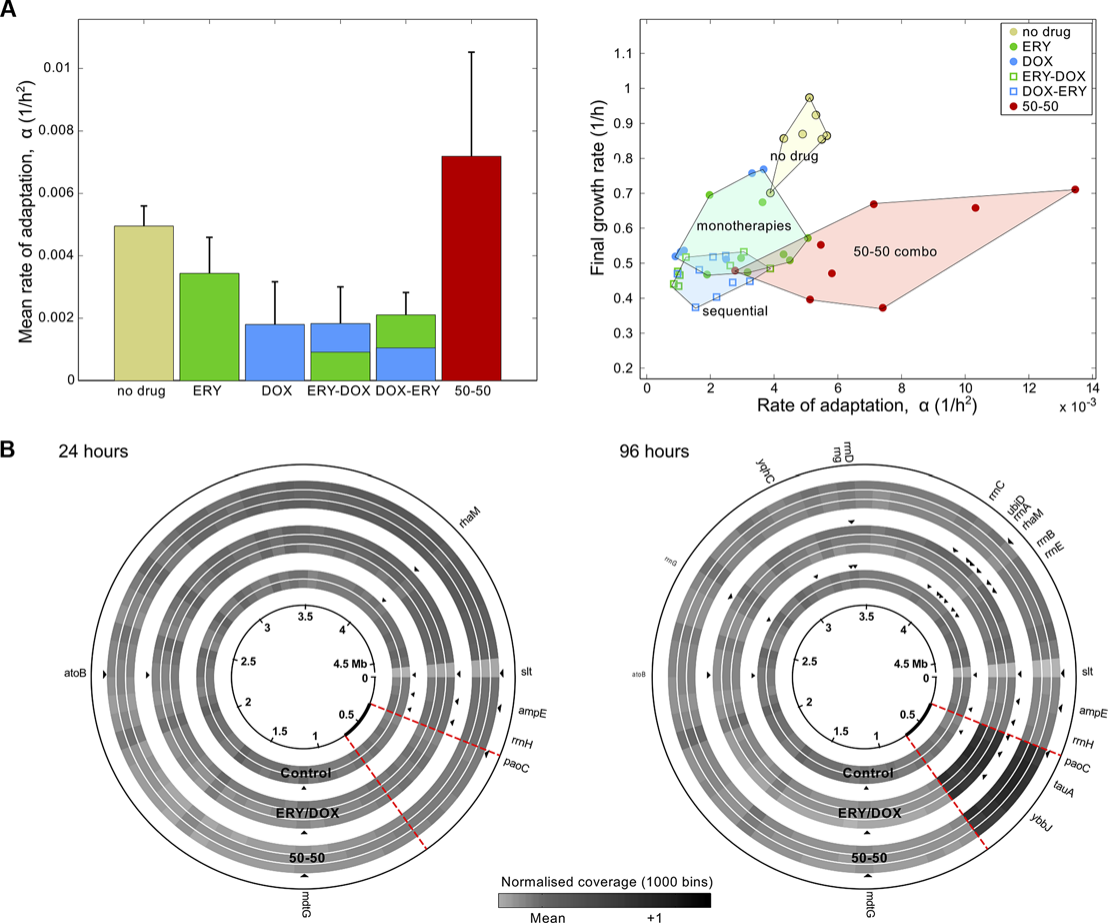
The rate of adaptation has a complex relationship with antibiotic dose. (A) Using IC_50_ dosages, growth rate adaptation (denoted *α* when defined in [14]) is greater for the 50/50 combination than for the EDEDEDED (“ERY—DOX”) and DEDEDEDE (“DOX—ERY”) sequential treatments. Adaptation can also be faster in the absence, rather than in the presence, of antibiotics. The right-hand plot shows each replicate separately (as a dot), indicating treatment clusters as coloured regions using the convex hulls of the datasets for each treatment type (whether no drug or single drug monotherapies, the 50/50 two-drug combination, or a sequential treatment). This shows sequential treatments minimise both final growth rate after eight seasons and the rate of adaptation. (B) Differences in the rate of adaptation in (A) are not accounted for by the duplication of the *acrRAB* operon, and sequential treatments do not prevent pump duplications. These coverage plots from sequenced populations at 24 h and 96 h show that both the combination (50/50) and sequential treatments (ERY/DOX) lead to the duplication of a genomic region from 273 Kb to 686 Kb that contains *acrRAB*. Left: the duplication was absent from all treatments after 24 h. Right: the duplication (the dark sector) is present in both the 50/50 combination and ERY—DOX sequential treatments after 96 h, but not in the no-drug control. Single nucleotide polymorphisms (SNPs) are highlighted as arrowheads next to the treatment in which they were observed. (S1 Data contains the data used in this figure.)

In order to determine genetic changes due to the differential stresses found in drug-free conditions and in the sequential and combination treatments, two treatments at IC_50_ that produced comparable densities at 96 h were subjected to a whole-genome sequencing analysis and compared to the drug-free populations (S1 Text, section 4). Writing “E” for a season of ERY and “D” for DOX, when metagenomes from the EDEDEDED and 50/50 combination treatments were sequenced, known resistance mutations were observed in both. Fig. 3B highlights a 412 Kb genomic region containing the *acrRAB* operon whose duplication was observed more frequently in both the combination and sequential treatments at 96 h (namely, eight seasons) than at 24 h (or two seasons; Fisher exact test for both, *p* = 0.05; Fig S17 in S1 Text, section 4). Treating sequentially does not, therefore, avert selection for duplications of the *acrRAB* operon.

We sought evidence for triplications of *acrRAB* by asking whether the ratio of coverage depths between amplified and nonamplified genomic regions was above a value of 2, the latter being the maximum value possible of this statistic if no triplications were present. However, at 96 h, in neither the sequential treatment (one-sided *t* test, *p*≈0.12,*T*≈1.68,*n* = 3) nor the 50/50 combination treatment (one-sided t-test, *p*≈0.061,*T*≈2.60,*n* = 3) was this value significantly above 2. Finally, single nucleotide polymorphisms (SNPs) were observed in the putative drug transporter gene *mdtG* (*yceE* [21]) in all conditions (Table S3 in S1 Text, section 4); this member of the *marA-soxS-rob* stress regulon mediates expression of the *acrAB-tolC* pump [22].

We expected the rate of adaption (defined as a rate of increase in growth rate [14]) to correlate positively with dose. Instead, we observed that adaptation can be just as rapid in the absence as in the presence of antibiotics (Fig. 3A), and our culture conditions may explain this. Slow growing cells, like persister phenotypes [23] and small colony variants [24], are cleared by our protocol, whereas cells that achieve rapid growth above approximately 6.6 generations every 12 h can survive. Rapid bacterial growth is associated with physiological changes that include negative DNA supercoiling and multiple DNA replication forks per cell [25], increased cell size [26], and heightened ribosomal demand [27]. The latter likely induced a stringent response in the fastest growth conditions (the absence of drug). In these conditions, SNPs associated with fatty acid degradation, lipid peroxidation stress, and sulphur transportation (*tauA*) were observed, the latter at high frequency (Table S3 in S1 Text, section 4). As *tauA* is expressed in our growth media only during cysteine limitation [28], overcoming *α*-amino acid starvation is a likely mechanism supporting the SNPs detected in all seven 23S ribosomal RNA operons (*rrn*) of *E*. *coli* by 96 h in the absence of drugs (Table S1 in S1 Text, section 4). Although mutations in the same *rrn* loci were observed at low frequency at 96 h in the slower-growing populations treated sequentially with drugs, none of these operons were mutated in populations treated with the drug combination. We hypothesise, therefore, that the antibiotics have slowed the rise and sweep of adaptive mutations needed for optimal growth in our culture conditions (Table S1 in S1 Text, section 4). Finally, we found no significant evidence of SNPs within drug targets in any conditions (S1 Text, section 4).

### Nonreciprocal Collateral Sensitivity

Antibiotic combinations are used to slow drug-resistance adaptation because they enhance their antibacterial effect through inhibitory synergisms [29] and because they reduce the number of potential resistance mutations. Here, consistent with prior studies of ERY—DOX combinations [13], growth rate adaptation is so rapid when using ERY and DOX in a synergistic IC_70_ combination that the bacterium is not cleared (Fig. 1A, Fig. 2B, Fig. 3A), and an analogous observation has already been made at double IC_95_ dosages [16].

Collateral sensitivities on the other hand, in which the prior use of one antibiotic sensitises the bacterium to the use of another, have few recognised mechanisms [30], but they too have been proposed as a possible basis for successful sequential treatments [7,31] because the change of environment hampers adaptation. Promisingly, the rate of adaptation is demonstrably lower here for sequential treatments than for combinations (Fig. 3A; p<10^-4^,*F*(2,21)≈16.8, one-way ANOVA with Bonferroni correction). However, it has also been suggested that the antibiotic sequences should follow optimised pathways through networks of drug choices that maximally sensitise the bacterium to treatment [7]. However, *E*. *coli* AG100 has pumps for both ERY and DOX, and we might therefore expect to observe cross-resistance, not collateral sensitivity, for this drug pair and this bacterium [6]. It therefore appears we do not have enough drugs for drug cycling to work in this model, but it will turn out, in fact, that we do.

This is because at least two cross sensitivity properties are observed. The first of these, which was noted recently for doxycycline [6], we term *nonreciprocal collateral sensitivity* (NCS), and it is defined as follows. Label two drugs “A” and “B” and choose equivalent dosages for both, meaning IC_*x*_ for some *x*, and let *D*(*T*_1_,*T*_2_) denote density of the population when treatment *T*_2_ follows treatment *T*_1_. We will also use the notation *A* ^*n*^ and *B* ^*n*^ to denote monotherapies with *n* rounds of treatment. Now, suppose we begin with a clonal population and treat with A for *n*+1 time units so that *D*(*A* ^*n*^,*A*) denotes the population density after the (*n*+1)-th treatment. Then, in a separate experiment, we treat with A for *n* time units followed by B for one time unit so that *D*(*A* ^*n*^,*B*) denotes the final population density. A nonreciprocal collateral sensitivity between A and B is said to occur when the switch from A to B results in a density decrease so that *D*(*A* ^*n*^,*B*)<*D*(*A* ^*n*^,*A*), whereas an analogous switch from B to A results in a density increase, meaning *D*(*B* ^*n*^,*A*)>*D*(*B* ^*n*^,*B*). When satisfied, this definition means A-adapted populations appear sensitised to drug B, whereas B-adapted populations have increased resistance to A.

If present, an NCS demonstrates that single-season inhibitory values cannot be used to infer the later inhibitory effect of antibiotics as the treatment proceeds; thus, IC_X_ values and rate of adaptation measures capture very different properties of the bacterium. For example, despite both drugs having equivalent inhibitory effects on a wild-type population after one dose, drug-resistance mutations could sweep more rapidly for one drug than the other, and this could result in an NCS. Nevertheless, if the observed collateral sensitivity is much larger than the cross-resistance within a dataset that indicates the presence of an NCS, appropriately chosen sequential regimens may still be sufficiently potent to eliminate the bacterium.

### Postulating a Mechanism That Supports Nonreciprocal Collateral Sensitivity

**Fig 4:**
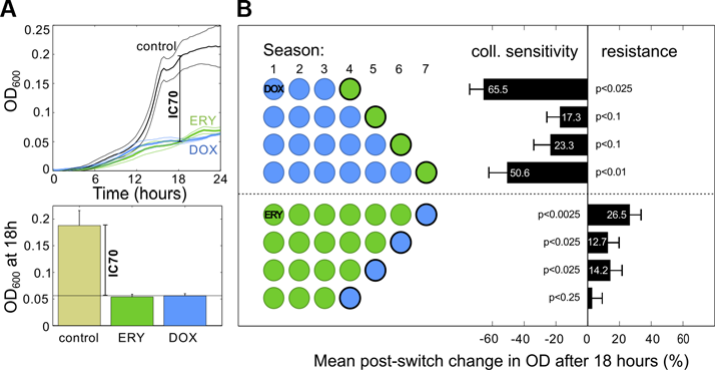
A nonreciprocal collateral sensitivity at I*C*_70_ dosages with respect to population densities. (A) The concentration of each drug was calibrated to ensure I*C*_70_ was achieved for both drugs, here at 18 h. (B) A nonreciprocal collateral sensitivity (NCS) determined using the (*n*+1)-protocol described in the text, where *n* = 3,4,…,7: the change in densities following a change in antibiotic demonstrates ERY → DOX cross-resistance but DOX → ERY cross sensitivity (*p*-values from *t* tests, *n* = 5). (S1 Data contains the data used in this figure.)

We first sought collateral sensitivities within the entire dataset shown in Fig. 2A but found no significant evidence (Fig S12 in S1 Text, section 3) that a switch from ERY to DOX had a different effect on population density than switching from DOX to ERY. We therefore tested for the presence of an NCS using a simpler “(*n*+1)-protocol”: *n* seasons of culture with one drug, followed by a switch to the other drug for just one season’s duration. This protocol (Fig. 4) shows that when AG100 is treated with ERY for *n* seasons (of 24 h duration) and DOX on the (*n*+1)-th season, both at IC_70_, the continued increase in bacterial density on the last season is consistent with cross-resistance (see Fig. 4B for *p*-values). However, when treating with DOX for *n* seasons and then ERY on the (*n*+1)-th, a density reduction is observed on the last treatment, consistent with a collateral sensitivity (Fig. 4B). This drug pair therefore possesses an NCS: although both inhibit growth of wild-type *E*. *coli* equally, they report different levels of inhibition on drug-adapted populations.

This observation accords with predictions of the following theoretical model [13]:

$$\frac{d}{dt}b_{1}=G(S,D_{1},E_{1})b_{1}-\delta (b_{1}-(1+\Delta )b_{2}),$$ (1)

$$\frac{d}{dt}b_{j}=G(S,D_{j},E_{j})b_{j}-\delta ((2+\Delta )b_{j}-b_{j-1}-(1+\Delta )b_{j+1}),$$ (2)

$$\frac{d}{dt}b_{N}=G(S,D_{N},E_{N})b_{N}-\delta ((1+\Delta )b_{N}-b_{N-1}),$$ (3)

$$\frac{d}{dt}S=-\frac{VS}{K+S}\sum_{j=1}^{N}b_{j},$$ (4)

$$\frac{d}{dt}D_{ext}=-d_{D}D_{ext}-\sum_{j=1}^{N}b_{j}\left(\varphi_{d}(D_{ext}-D_{j})-\frac{v_{d}p_{j}}{k_{d}+p_{j}}D_{j}\right),$$ (5)

$$\frac{d}{dt}D_{j}=-d_{D}D_{j}+b_{j}\left(\varphi_{d}(D_{ext}-D_{j})-\frac{v_{d}p_{j}}{k_{d}+p_{j}}D_{j}\right),$$ (6)

$$\frac{d}{dt}E_{ext}=-d_{E}E_{ext}-\sum_{j=1}^{N}b_{j}\left(\varphi_{e}(E_{ext}-E_{j})-\frac{v_{e}p_{j}}{k_{e}+p_{j}}E_{j}\right),$$ (7)

$$\frac{d}{dt}E_{j}=-d_{E}E_{j}+b_{j}\left(\varphi_{e}(E_{ext}-E_{j})-\frac{v_{e}p_{j}}{k_{e}+p_{j}}E_{j}\right),$$ (8)

where *j* = 2,…,*N*-1 is a parameter that controls the number of efflux genes each cell can express. Equations 1–8 capture the densities, *b*_*j*_, of bacteria with duplications of a gene that exports drugs from within the cell. At time *t*, *S* is the concentration of a limiting carbon source, *D*_*ext*_ and *E*_*ext*_ are extracellular concentrations of each drug, DOX and ERY respectively, *D*_*j*_ and *E*_*j*_ are the intracellular drug concentrations, and drugs degrade at rates *d*_*D*_ and *d*_*E*_. The variable *p*_*j*_ represents the expected number of efflux pumps expressed by a cell with *j*-1 efflux genes, for each *j*≥2. We also assume that *p*_1_ = 0 so that it is possible for cells to encode the pump without expressing it (meaning genotypes for which *j* = 1 have the efflux gene but do not express it). More generally, the nonzero quantity *p*_*j*+1_ is defined, for *j*≥1, by *j*/(1+*γ*∙*j*)), and then *p*_*j*_/(*k*_*e*_+*p*_*j*_) is the probability that a given drug molecule is bound to an efflux pump. This model is a simplification of the competition each transcription unit has for each efflux operon (the *acrRAB* promoter), whereby a diminishing return is present in the number of pumps expressed as the number of efflux genes increases; the rate of diminishing returns is controlled by *γ*>0. Increases and decreases in the number of efflux genes in a cell are assumed to be a Poisson process with parameter *δ* per cell per hour.

Other variables in Equations 1–8 have the following meaning: *φ*_*e*_,*φ*_*d*_ are antibiotic diffusion rates across the cell membrane, *ν*_*e*_,*v*_*d*_ are maximal drug efflux rates, and *k*_*e*_,*k*_*d*_ are half-saturation constants associated with pump-antibiotic binding; *V* and *K* are maximal uptake rate and half-saturation constants associated with a Michaelis-Menten uptake model of the limiting carbon source (*S*); growth rate *G*(*S*,*D*,*E*) = *cVS*/((1+k_e_E+*κ*_*d*_ *D*+*κ*_*ed*_ *ED*)(*K*+*S*)) is proportional to uptake rate via a per-sugar biomass yield constant *c*, and *G(S, D, E)* is reduced in value synergistically by the drugs (where *κ*_*e*_,*κ*_*d*_ and *κ*_*ed*_ are parameters that control drug efficacy and strength of synergy); *δ* is the rate of amplification of the efflux gene, and *δ*(1+Δ), a value necessarily greater than *δ*, is the rate of loss of the gene. *N*-1 is the maximum number of copies of the efflux gene. We set *N* = 3 to represent three different cell phenotypes: an unexpressed pump gene (a wild type), a single expressed pump gene, and one additional copy of that gene in which both copies are expressed. Finally, the model is simulated with several seasons so as to mimic the in vitro protocol, with the loss of 99% of all cells implemented at the end of each season. Equations 1–8 were solved numerically using a parameterisation determined from a prior training dataset (S1 Text, section 5) [13].

**Fig 5:**
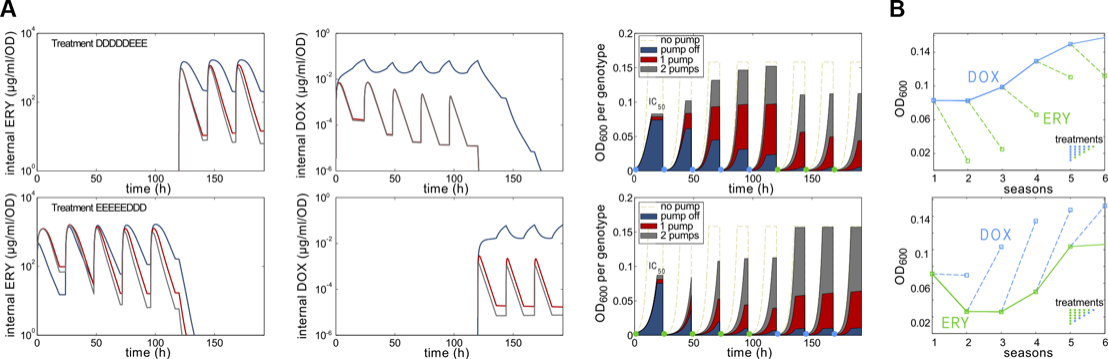
A mathematical model indicates frequency-dependent selection for the pump duplication can cause a nonreciprocal collateral sensitivity profile with respect to population densities. (A) The first two columns indicate modelled internal drug concentrations in three cell phenotypes (dark blue: “wild-type” cells not expressing the pump; red: the pump gene is expressed; dark grey: two pump genes are expressed). The third column shows modelled population densities through time, indicating the frequencies of each phenotype within that density. The different drugs select for resistant (pump-expressing) and susceptible (pump-not-expressed) phenotypes at different rates, despite both having been calibrated to equal inhibitory effect (namely I*C*_50_) on a population consisting almost exclusively of wild-type cells by the end of day 1. These simulations show that monotherapies consisting of either drug select for different population structures, each having different frequencies of the pump gene and its duplication, depending on which drug is being applied. Thus, given n days of adaptation to DOX followed by adaptation to ERY, after the switch, density decreases. (B) Implementing the (*n*+1) protocol in the model is consistent with the data of Fig. 4. (S1 Data contains the data used in this figure.)

Although our theory does not capture all aspects of our data, computations show that, like *E*. *coli*, the model possesses an NCS (Fig. 5). The model predicts that a pump asymmetry due to different efflux efficiencies of ERY and DOX produces populations with differential susceptibility to each drug resulting from having different frequencies of drug-susceptible wild-type cells existing in mutation-selection equilibrium with less susceptible mutants (Fig. 5A). Supporting the hypothesis of different efflux efficiencies of ERY and DOX, data from the *E*. *coli acr* efflux knockout strain AG100A(Δacr) (Table S2) [13] shows that the loss of *acrB* reduces the IC_50_ of ERY to approximately 5% of the wild-type AG100 value but reduces it to just 23% in the case of DOX. The model captures others features of the data, particularly that appropriately chosen sequential treatments produce fewer bacteria than the combination, yet some sequential treatments produce more (Fig S18 in S1 Text, section 5).

**Fig 6:**
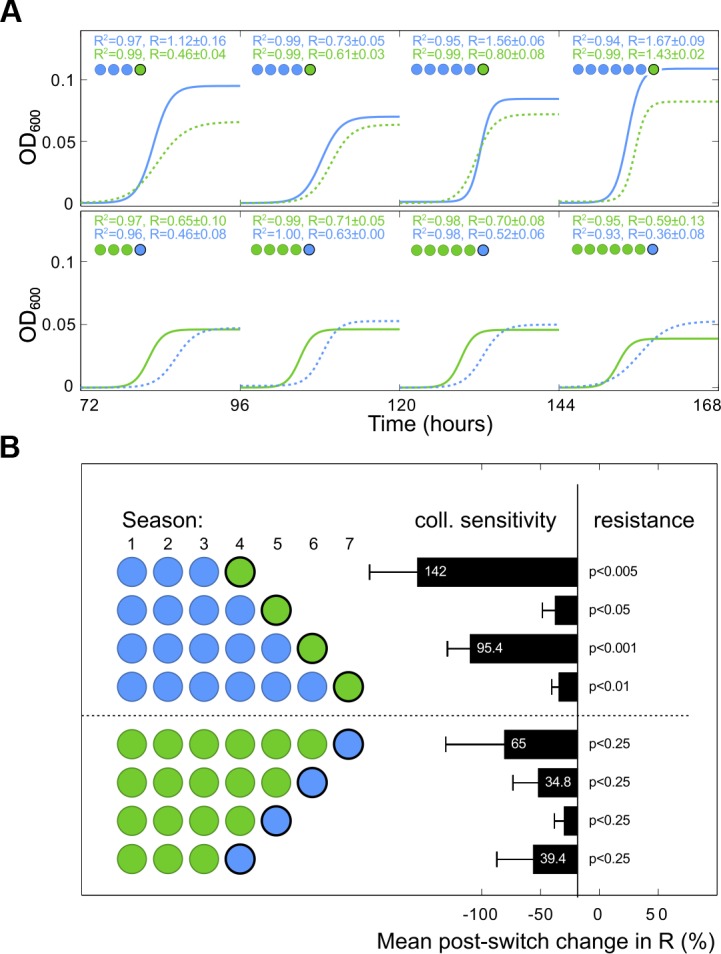
A collateral sensitivity at IC_70_ with respect to per capita growth rate, R. (A) Optical density time series data used in Fig. 4 was reused by fitting a logistic growth model (defined in the text) to estimate growth rates. For clarity, both the growth rate parameter *R* and the regression coefficient *R* ^2^ from exemplar fits are indicated alongside modelled dynamics. (B) The resulting dataset shows significant and nonsignificant collateral sensitivities with respect to growth rate (*R*) following an exchange of antibiotic (*t* tests, *n* = 5). (S1 Data contains the data used in this figure.)

A second cross sensitivity property of the ERY—DOX was also established, as follows. Having found a mechanism for an NCS with respect to population densities, we hypothesised that the (*n*+1)-protocol data could exhibit cross sensitivities with respect to other measures of bacterial fitness. To demonstrate this, we fitted the logistic growth model $\frac{dx}{dt}\;=\;R\;\cdot \;x(1\;-\;x/K)$ to bacterial density time series, where the parameter *R* is per hour per capita growth rate and *K* is the population carrying capacity. The resulting data exhibits collateral sensitivities irrespective of the order in which the drugs were exchanged because a reduction of *R* was observed following a change of drug for every *n* tested (from 3 to 6), although not all reductions were significant (Fig. 6).

## Discussion

Despite the ability of bacteria to adapt to an antibiotic challenge, our laboratory model shows that one can exploit a sensitising property of fluctuating environments to eliminate a bacterium eventually at dosages that only inhibit growth by 70% initially. However, ours is a very simple treatment model inspired by bacterial infections, and we do not wish to overstate its predictive power in relation to the treatment of humans. In particular, the loss of slow-growing cells from our microcosm is not representative of the in vivo conditions in which slow-growing, antibiotic-tolerant phenotypes can be responsible for recalcitrance to treatment [32]. Our model is also limited because it lacks an immune response or any of the environmental complexity found in the human body.

The clinical practise of how antibiotics are used to treat bacterial infections begins with the minimal inhibitory concentration (MIC), the minimal drug concentration at which no visible growth of a bacterium is observed after overnight culture in vitro [33,34]. After determining the MIC, antibiotics are deployed at high enough dosages so that peak concentrations are achieved in vivo well in excess of this number [35]. Experience has shown that super-MIC dosages are necessary for successful recovery from infection when using combination treatment and monotherapy [33–39], although there is recent evidence of effective lower-dose antifungal, anti-MRSA (methicillin-resistant *Staphylococcus aureus)*, and antimalarial treatments in vivo [40–42]. However, so few sequential treatments have been trialled in the clinic that there is little accumulated data on or understanding of what dosing or scheduling criteria might be needed when using sequential treatments in vivo.

Furthermore, not all IC_70_ sequential treatments lead to clearance, and some drug sequences produced higher population densities than the equivalent combination treatment (Fig. 2A). The reasons for this are not clear, although both data and the theoretical efflux model do exhibit large between-treatment variation (Fig. 2A, Fig S18 in S1 Text, section 5). A prior hypothesis states that greater antibiotic heterogeneities in the bacterial environment should diminish the rate of drug-resistance adaptation [43]. This would mean, for example, that bacteria adapt more readily to an EEEEDDDD treatment (one drug switch) than to EDEDEDED (seven switches). We therefore sought a relationship between bacterial population densities and the number of drug switches implemented in different sequential treatments but found no evidence of the predicted correlation (Fig S11 in S1 Text, section 3).

It is well known that the use of low dosages can select for resistant strains when they are competed in co-culture with susceptible strains [17]. However, that mid-dose clearance is still possible has a simple explanation in principle: the antibiotic sequences eventually reduce, and then maintain, Malthusian fitness of the evolving population below zero. To examine this in the simplest of theoretical contexts, suppose *B*_*n*_ represents bacterial population density after *n* rounds of treatment where 1-*I*, with 0<*I*<1, is an expected fraction of cells that are not cleared by the treatment but instead lost because of other effects (for example, host immunity) each treatment. Indeed, granulocyte-mediated clearance has been shown to achieve a two-log_10_ reduction in bacterial load in a 24 h period using a murine model [44], giving a value of *I*≈1/100.

At any sublethal dosage, whereby exponential population growth (at rate *r*) occurs between treatments spaced *T* time units apart (cf. Fig. 1A), it follows for a bacteriostatic antibiotic at a dose of “*A* units” that *B*_*n*+1_ = *I*∙*B*_*n*_ *e* ^(*r*-*A*)*T*^. Bacterial clearance is assured when the population decays eventually; this happens when *B*_*n*+1_/*B*_*n*_<1, which is equivalent to the condition *A*>*r*-(log(*I* ^-1^))/*T*. Note that this value is less than *r* by an amount that depends on *I*. If we now define an analogy of the MIC as, say, the IC_99_ in this toy model, which is the antibiotic dose that reduces bacterial growth by 99% after one treatment, the condition on *A* to achieve IC_99_ is *e* ^(*r*-*A*)*T*^<1/100, or *A*>*r*+(log 100)/*T*. This value is greater than *r* and therefore effective therapeutically, but it is not representative of the critical minimal dose needed to clear the bacterium [45].

The absence of visual eradication overnight in vitro should not, according to this argument, itself be used as a rationale to preclude the practical use of an antibiotic drug. Indeed, the antifungal azole drugs are used to treat *Candida albicans* clinically at dosages that do not always eliminate population growth after overnight culture in vitro [45–47]. In our in vitro study, we needed to keep the rate of adaptation low for the above theoretical rationale to work, and only certain sequential treatments were able to do this (Fig. 3A and 3B). The requirement for low rates of adaptation likely needs the mutations and physiological changes that arise early during treatment, when population sizes are large, to provide no benefit, or even be deleterious later during treatment and so prevent recovery when population size, and mutational supply, is small.

There have been clinical successes for one particular sequential treatment: *Helicobacter pylori* infection has improved eradication rates for a sequential treatment relative to a combination therapy at the same dose [8,9], although geographical variations in successes have been observed and attributed to pathogen strain differences [48]. We hypothesise that the treatment of other clinical pathogens may be possible using sequential antibiotic treatments.

We note that low dosing is used to treat some bacterial infections. The ability of antibiotics to act as modulators of gene expression at low doses [49] can be exploited, for example when certain drug classes (including macrolides) are used to control the expression of virulence factors in MRSA [50]. Moreover, the use of *β*-lactam antibiotics as a low-dose adjuvant is a novel strategy in the treatment of recalcitrant MRSA infection [51,52], even though MRSA is resistant to most of these drugs. The *β*-lactam does not target the cell directly; rather, it enhances the activity of host peptides that are not antimicrobial per se but which modulate the host immune response [53].

However, it is not our intention to advocate for the indiscriminate clinical use of low-dose regimens. Rather, we are claiming that sequential dosing strategies exist for administering antibiotics that are sufficiently potent, and which prevent adaptation enough, to clear a bacterium when the equivalent dose combination treatment fails to do so. That this can be done even though the bacterium has a scaleable multidrug resistance mechanism in its chromosome gives us cause to hypothesise that new ways of optimising antibiotic use in vivo can be found by alternating them as part of treatment.

## Materials and Methods

### Media and Strains

We used *E*. *coli* AG100 (a gift from Stuart B. Levy) and M9 minimal media (0.2% glucose and 0.1% casamino acids). Stock solutions of DOX and ERY were made from powder stocks (Sigma-Aldrich) at 5 mg/ml in water for DOX and 100 mg/ml in ethanol for ERY and stored at -20°C. All subsequent dilutions were made from these stocks and kept at 4°C.

### Batch Transfer Protocol

A microtitre plate reader measured optical densities every 20 min at 600nm as a proxy for population densities in different environments (R^2^>0.99, Fig S1 in S1 Text, section 1). 96-well plates containing 150 μLof liquid per well incubated at 30°C were used to culture bacteria; these were shaken in a linear manner before each measurement was taken.

For prolonged exposure to antibiotics, inoculating bacteria were taken from one colony and cultured overnight in M9 minimal media (0.2% glucose, 0.1% casamino acids) at 30°C in a shaker-incubator. At the end of each season, a 96-pin replicator sampled the liquid volume, which was then transferred to a new plate containing fresh growth medium and antibiotics; the same environment for each replicate population was maintained. Every subsequent transfer was performed using the 96-pin replicator; the volume transferred was approximately 1.5μL. OD time series were imported into Matlab R2013b to subtract the background (blank wells containing only medium) and generate all other statistics.

### Live Cell Counts

No claim is made on the basis of optical density data alone that a zero population density had resulted from treatment. Zero densities were determined by observing an OD value below 10^-2^ units, whereafter the presence of cells was determined by spot tests. Serial dilutions were then used to determine live cell numbers in colony-forming units, if any were detected (Fig S15 in S1 Text, section 3).

### WGS Data Accession Number

Whole-genome sequence data with 18 samples and an annotated draft genome is available from the European Nucleotide Archive (ENA) with study accession number PRJEB7832.

This data can be downloaded from http://www.ebi.ac.uk/ena/data/view/PRJEB7832.

## Supporting Information

S1 Data This file contains the data used to produce all figures in the main text. (XLSX)

S2 Data This file contains the data used to produce all figures in S1 Text. (XLSX)

S1 Text Supplemental information. Contains experimental materials and methods, typical growth data and the rate of adaptation, additional data, a whole-genome sequencing analysis, an additional simulation of the mathematical model, and Figs. S1–S18, Tables S1–S6, and their references.
(PDF)

## Abbreviations

DOX: doxycycline
ERY: erythromycin
IC_x_: inhibitory concentration (with an x% degree of inhibition)
MIC: minimal inhibitory concentration
MRSA: methicillin-resistant *Staphylococcus aureus*
NCS: nonreciprocal collateral sensitivity
OD: optical density
*rrn*: ribosomal RNA operon
SNP: single nucleotide polymorphism
