# Supplementary material for: Using a Sequential Regimen to Eliminate Bacteria at Sublethal Antibiotic Dosages
Source: PLoS Biol. 2015 Apr 8;13(4):e1002104. doi: 10.1371/journal.pbio.1002104 (PMC4390231; doi:10.1371/journal.pbio.1002104)
Supplement: S1 Text — Contains experimental materials and methods, typical growth data and the rate of adaptation, additional data, a whole-genome sequencing analysis, an additional simulation of the mathematical model, and Figs. S1–S18, Tables S1–S6, and their references. (PDF) [file pbio.1002104.s003.pdf]

S1 TEXT: USING A SEQUENTIAL REGIMEN TO ELIMINATE BACTERIA AT SUB-LETHAL ANTIBIOTIC DOSAGES

Ayari Fuentes-Hernandez[+], Jessica Plucain[+], Fabio Gori[+], Rafael Pena-Miller,
Carlos Reding, Gunther Jansen, Hinrich Schulenburg, Ivana Gudelj and Robert Beardmore[*]

§1 Experimental Materials and Methods

1.1 Media and strains.

All experiments were conducted using *Escherichia coli* K12(AG100) and M9 minimal media (0.2% glucose and 0.1% casamino acids). We used two antibiotics with a synergistic interaction: *doxycycline* (DOX) and *erythromycin* (ERY). Stock solutions of antibiotics were made from powder stocks (Sigma-Aldrich) at $5mg/ml$ in water for DOX and $100mg/ml$ in ethanol for ERY and stored at $-20°$C. All subsequent dilutions were made from these stocks and kept at 4°C. After testing, no measurable decay in the efficacy of the antibiotic has been observed when storing these antibiotics in either of these conditions for one week, or less.

1.2 Batch-transfer protocol

For all the protocols consisting of multiple serial batch transfers referred to in the main text, with each transfer conducted once per 'season', we used the same microtitre plate reader (BioTek) to measure optical densities every 20 minutes at $600nm$ as a proxy for bacterial population densities in different environments (written as OD_{600nm} or just OD). We used 96-well plates containing $150\mu L$ of liquid in each well incubated at 30°C to culture bacteria, shaken in a linear manner before each OD measurement was taken.

For an experiment exposing bacteria to antibiotics lasting several seasons (where each season lasts 12h or 24h) an initial inoculation was performed using an isogenic population obtained from a single colony and cultured overnight in M9 minimal media (0.2% glucose, 0.1% casamino acids) at 30° in a shaker-incubator. At the end of each season, the same 96-pin plate replicator was used to sample the liquid volume (containing bacteria and spent medium) which was then transferred to a new plate containing fresh growth medium and antibiotics, ensuring the same environment for each replicate population was maintained. Every subsequent transfer to a fresh plate containing medium was performed using the same 96-pin replicator, we estimated the volume transferred to be $1.5\mu L$. The so-obtained, time-dependent optical densities were then imported into Matlab in order to subtract the background (determined from blank wells containing only medium) and compute the mean optical densities and other statistics.

N.B.: media-only wells testing for the presence of potential contamination were used on every 96-well microtitre plate. If any showed turbidity above blank levels, the assay in question was repeated.

1.3 Live cell counts: optical density is a reasonable proxy for cell density

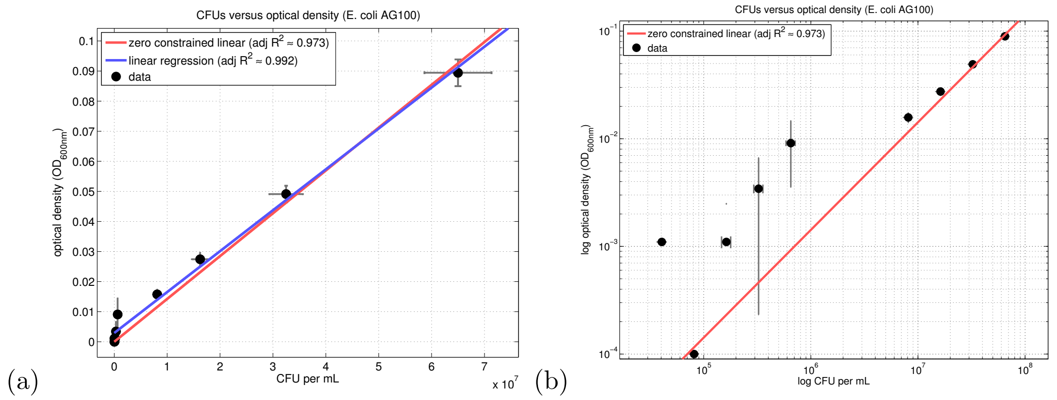

Fig. S1 – Data showing that OD is positively correlated with a live cell count (CFU per ml) in the plate reading devices that we use. (a) *E. coli* K12(AG100) was cultured in M9 and serially diluted to different OD values, these were then counted to reveal CFU values (all error bars are 95% confidence intervals of the mean, three replicates). A linear regression (blue) is shown next to data. A constrained linear regression that must pass through the datapoint $(0, 0)$ is also shown (red). (b) The latter indicates that OD has the potential to over-estimate live cell numbers at the lowest densities.

Many of the bacterial growth and inhibition experiments described in this article require the continual measurement of bacterial population densities and this cannot be done with by-hand lab techniques, such as colony counting. We therefore use devices for which proxies of population density can be rapidly produced using automated protocols that read densities as light absorbance.

No claim is made in the article on the basis of OD data alone that a zero population density has resulted under any treatment conditions, the measurement error inherent to such devices is too great for that to be reliable. When any claim is made that no cells are present, this is always deduced from first observing an OD_{600} value of below 10^{-2} units after at least 12h growth, thereafter cells are counted manually following a spot test in order to determine whether cells are present and, if so, how many. Fig. S1 shows that OD is a reasonable indicator of bacterial population density: this measure correlates positively, and indeed linearly, with viable cell counts (measured in units of colony-forming units per ml).

1.4 Dose-response curves

We determined dose-responses by measuring bacterial OD_{600nm} dynamics at different dosages of both antibiotics, the resulting growth data was used to estimate the drug concentration necessary to achieve $x\%$ inhibition with respect to the null-antibiotic control (at drug concentrations hereafter denoted IC_x). As illustrated in Fig. S2, the approximate IC_{50} values obtained for a 12h experiment were $0.04\mu g/ml$ for DOX and $6.13\mu g/ml$ for ERY. The degree of variation in these measures is illustrated using a 95% confidence interval for each dose response curve in Fig. S2.

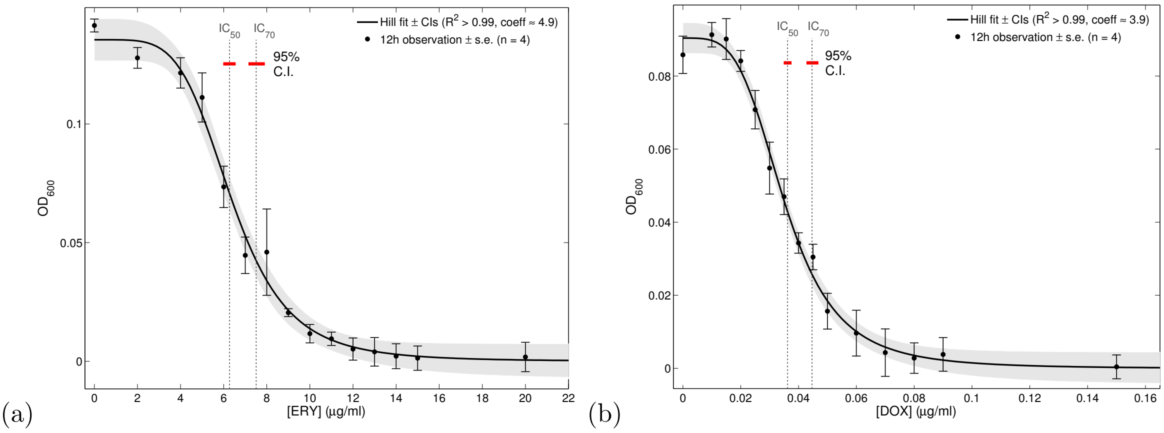

Fig. S2 − 12h dose-response curves for *E.coli* K12(AG100) for both drugs used in this study: (a) erythromycin and (b) doxycycline. Superimposed Hill function datafits are used to estimate the value of, and error in, x when computing IC_x values, the shaded region represents an estimated 95% confidence interval, Hill coefficients are stated in the top-right corner of each datatfit (4 replicates). Red lines denote the 95% confidence intervals of IC_{50} and IC_{70}, as the lines do not intersect estimates of both parameters are significantly different.

1.5 Drug interaction curves

We say that synergy is exhibited by a drug pair, at a given time, if the following holds. If $B(t, D, E)$ denotes the time-series of bacterial densities as a function of time and dose, given two drugs denoted D and E, then when

(1) $B(t_0, D_0, 0) = B(t_0, 0, E_0)$ (an equi-potency assumption on both drugs)
(2) $i(\theta) := B(t_0, \theta D_0, (1-\theta)E_0) < B(t_0, 0, E_0)$ for all θ between 0 and 1, (note the later equals $B(t_0, D_0, 0)$ by assumption)

the drugs D and E are said to synergise at basal dosages D_0, E_0 at time t_0. Under these assumptions, by fitting a quadratic to the interaction function $i(\theta)$, so that $\alpha\theta^2 + \beta\theta + \gamma \approx i(\theta)$, we can determine the numerical degree of synergy from the value of α. When the inequality $\alpha > 0$ holds for all E_0, D_0 and t_0, a criterion that can be established in practise using standard numerical fitting routines and a t-test, we then say D and E synergise. This procedure, called the α-test, is detailed fully in [18]. The quadratic fits to bacterial density data in Fig. S3 establish that this statistical test is passed for ERY, DOX in the experimental described conditions described above using the strain *E.coli* K12(AG100).

1.6 The $(n+1)$-protocol: measuring collateral sensitivity and cross resistance

In order to quantify how acclimation (UK English equivalent 'acclimatisation') to one antibiotic impacts population growth after a switch to another antibiotic occurs, and to measure this as a function of the duration of the acclimation period, we evolved five replicates of an isogenic population of *E. coli* for $n+1$ seasons at IC_{70} (see Fig. S4), where each season lasts 24h, as follows.

Bacteria were exposed to a constant environment (DOX or ERY) for $n \geq 3$ seasons before being transferred into an environment containing the other drug: those growing in DOX were transferred into an environment containing only ERY, those growing in ERY were then treated with DOX. We compared the optical densities of the population that underwent a change in drug at the end of the n-th season to the corresponding optical density of the population growing under constant conditions. This allows us to determine whether acclimation to one drug is associated with increased sensitivity to the other.

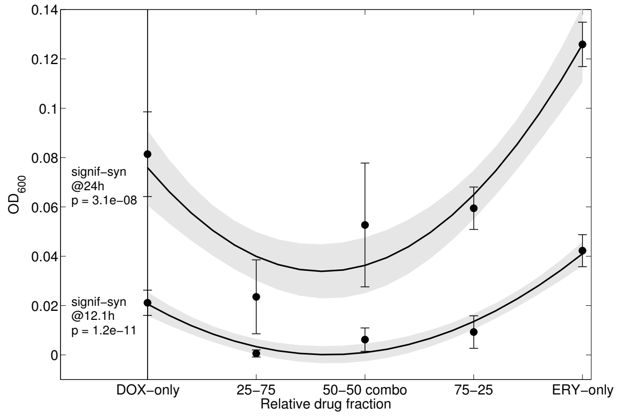

Fig. S3 – Establishing statistically significant synergy at both 12h and 24h using the α-test for synergy defined in [18] (p-values indicated in the figure) with both erythromycin and doxycycline at their respective IC_{50} dosages. Superimposed fits of a quadratic function is shown, the shaded region represents an estimated 95% confidence interval. The property that the fit is a convex function (and not concave) establishes the ERY-DOX synergy because a 50-50 combination reduces the optical density (indicated on the y-axis) relative to both ERY-only and DOX-only monotherapies.

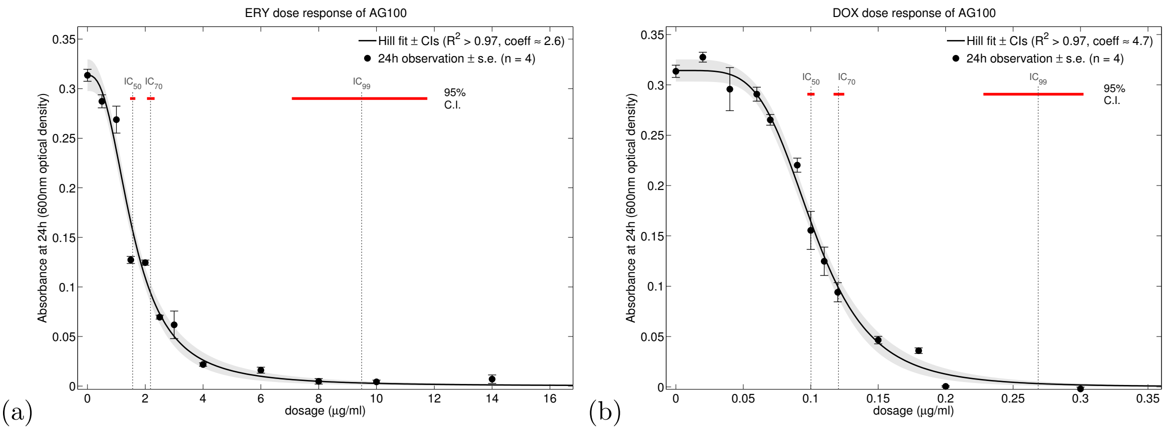

Fig. S4 – 24h dose-response curves for *E.coli* K12(AG100) for both drugs used in the $(n + 1)$-protocol: (a) erythromycin and (b) doxycycline. (To ensure stationary phase occurred beyond 12h, the M9 medium was supplemented here with a three times greater concentration of glucose than Fig. S2.)

The treatments implemented using the $(n + 1)$-protocol are illustrated in Fig. S5.

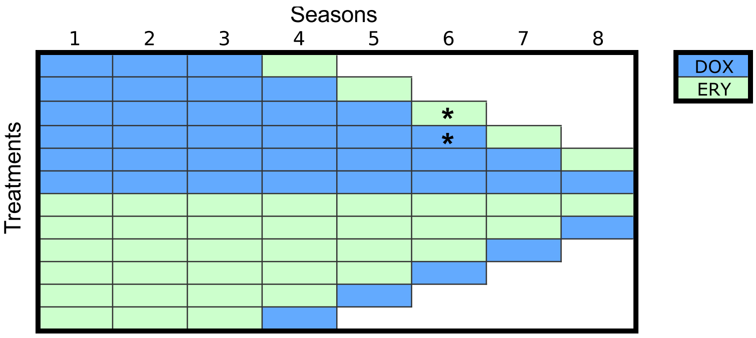

Fig. S5 – Treatments used in the $(n + 1)$-protocol. First, bacteria are grown under the effect of a single-drug (ERY in green and DOX in blue) for n days until a switch is performed and the other antibiotic is used for one day.

1.7 The sequential deployment of antibiotics.

Differences in population densities produced by different sequential treatments were measured as follows. An 8-season batch transfer protocol was implemented, with each season having 12h duration. At the end of each season, a 1% sample of the final population was transferred using a pin replicator into a fresh 96-well plate containing a replenished environment (containing both fresh M9 media and antibiotic).

Drug treatments consisted of the sequential deployment of DOX and ERY whereby only one drug was used within each season. The order in which the drugs were deployed defined each treatment and, in order to perform a like-with-like comparison, we introduced the constraint that all treatments were balanced: by the end of an 8-season treatment, each drug would be used for 4 seasons (ERY for 4, DOX for 4) and this is true for all treatments implemented. Thus, these sequential treatments are illustrated using the schematic in Fig. S6. Each such treatment was initially replicated three times.

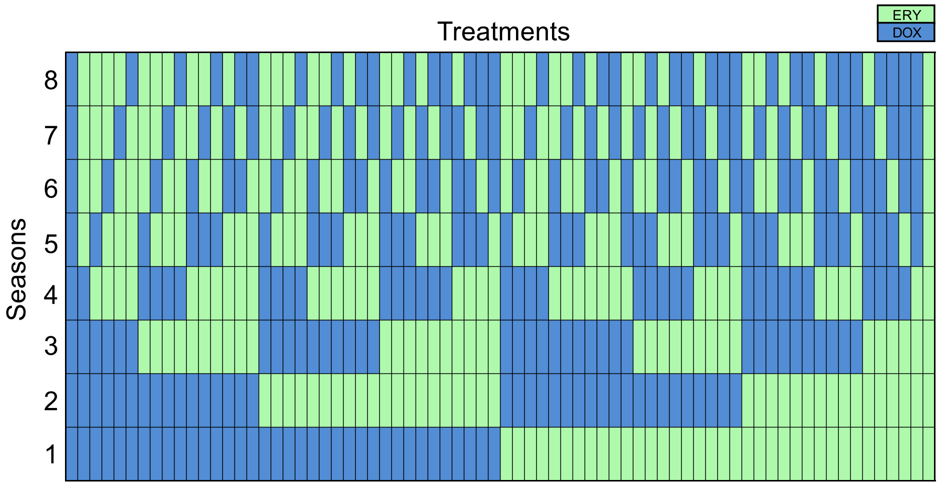

Fig. S6 – Sequential treatments and monotherapies implemented in an 8-season evolutionary assay (ERY in green, DOX in blue). All treatments are balanced: they have an equal number of seasons for which each drug is deployed.

Different results are obtained in this protocol depending on the choices of dose for ERY and DOX. All treatments, including monotherapies (*ie.* treatments that use one antibiotic for the entire duration), were implemented at IC_{50} and IC_{70} dosages. As a control, we also implemented the 50-50 combination treatments where, to clarify what this means, if monotherapies used dosages of $3\mu g/ml$ for ERY and $0.1\mu g/ml$ of DOX, the 50-50 treatment would have $\frac{1}{2} \times 3\mu g/ml$ of ERY and $\frac{1}{2} \times 0.1\mu g/ml$ of DOX. By design, the 50-50 combination treatment must yield a lower OD than the monotherapies in the first season to be consistent with previous studies reporting ERY and DOX (and our prior data) as a synergistic drug pair [9, 18].

§2 Typical growth data and the rate of adaptation

Typical raw data typical are shown in Fig. S7. If no drug is deployed (yellow data) the OD increases each day with the bacterium entering stationary phase progressively earlier each transfer (a.k.a. season). Antibiotic use reduces population growth and delays entry into stationary phase.

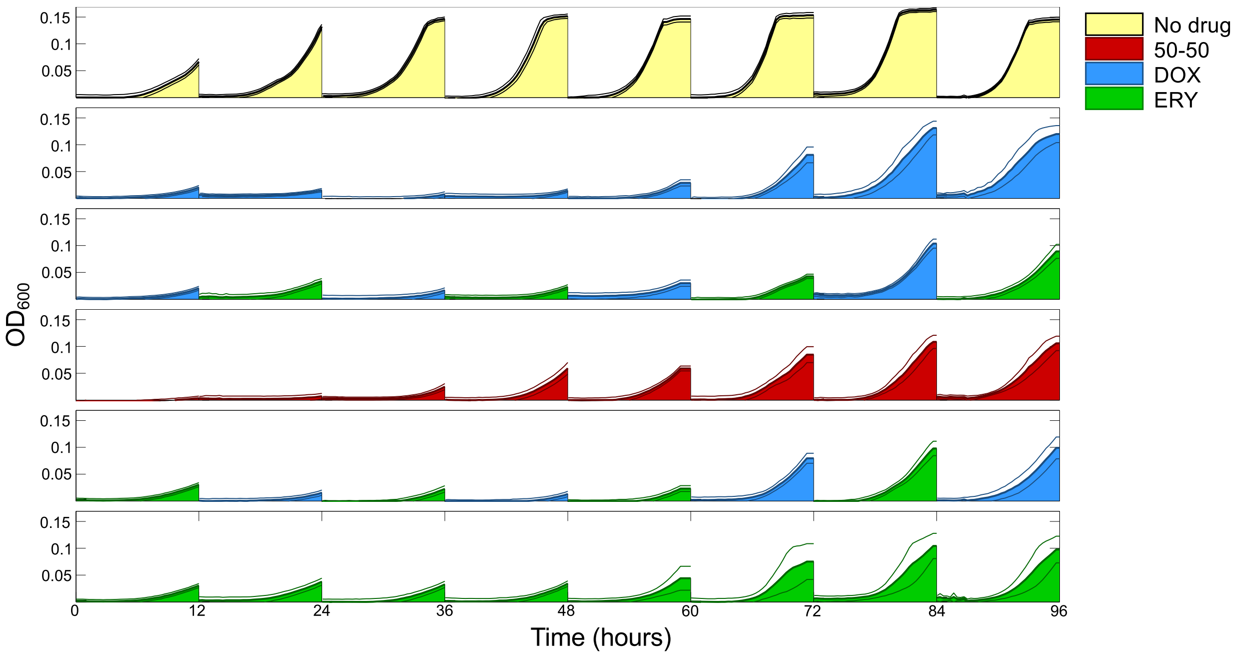

Fig. S7 – Mean optical density data (plotted as coloured areas) in an 8-season experiment with transfers performed every 12 hours. Each row represents a different treatment and the drug used each season is colour-coded (yellow areas represent no drug, blue the use of DOX, green the deployment of ERY and red a 50-50 combination of DOX and ERY). Solid coloured lines denote s.e., $n = 5$.

Calculating the phenotypic rates of adaptation using the measure defined in [9] for the data presented in Fig. S7 produces Fig. S8. This shows that growth rate adaption to treatment is different for each condition, being fastest for the 50-50 antibiotic treatment and slowest for the DOX monotherapy. Interestingly, the rate of adaptation for populations cultured in no-drug conditions are between these two extremes. This data shows that antibiotic inhibitory effect and the rate of adaptation are not necessarily correlated and that adaptation to a media-only environment (*i.e.* no drug) can be just as rapid as adaptation to antibiotic-inhibited growth.

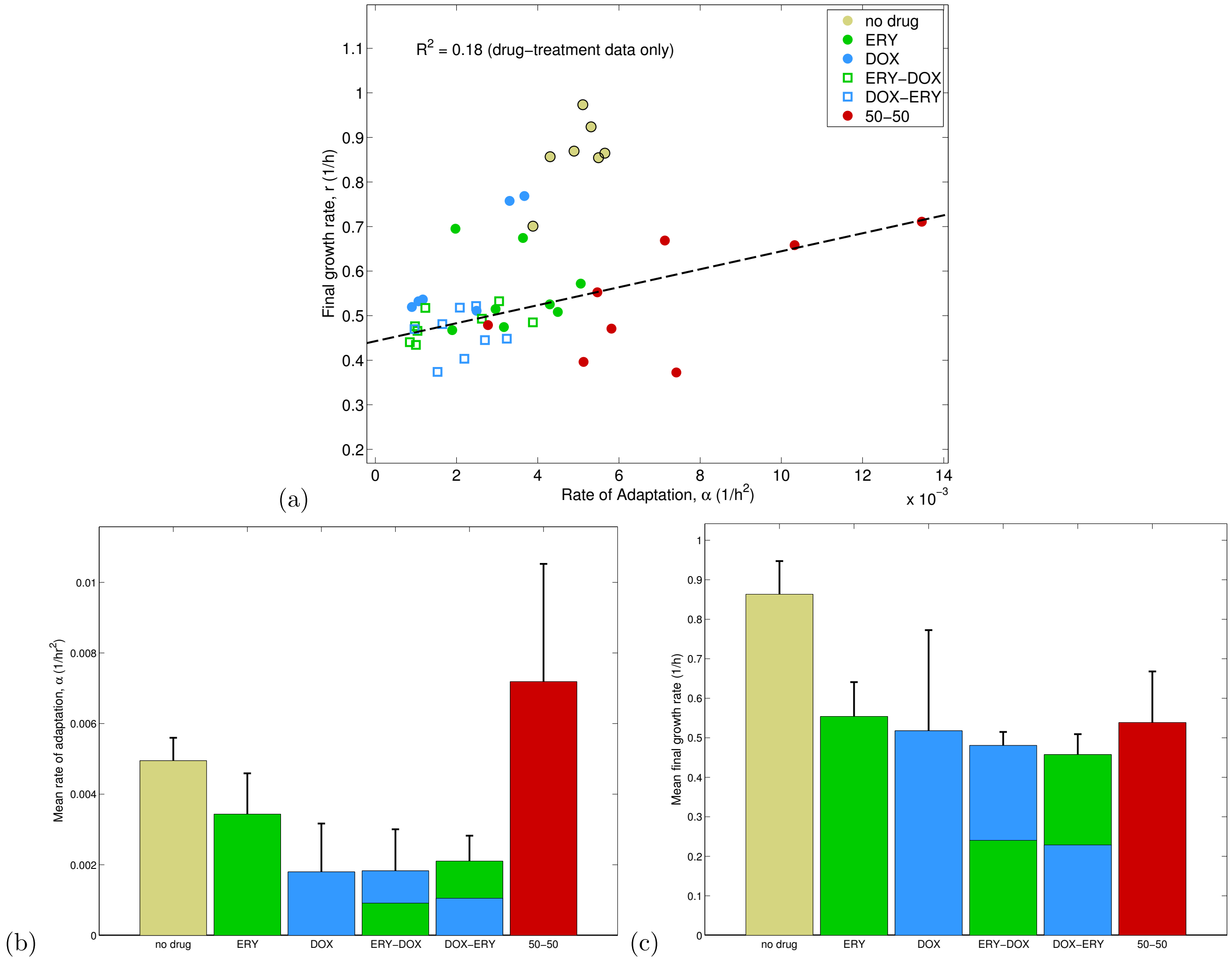

Fig. S8 – (a) The rate of adaptation for each of the treatments shown in Fig. S7. There is a positive correlation between rate of adaptation and final growth rate in the treated populations. The no-drug conditions produce higher growth rates than the drug-treated conditions and adaptation is just as rapid as in drug-treated conditions. (b) Adaptation is fastest to the synergistic 50-50 combination treatment. Importantly, the rate of adaptation to the no drug control environment is not the lowest of all the conditions tested. This data indicates no positive correlation between rate of growth adaptation and the inhibitory effect of the antibiotics because adaptation can be just as fast in the absence, as in the presence, of antibiotics. (c) Growth rate is, of course, greatest in the last season in the populations with no antibiotic. Moreover, growth rate is *not lowest* on the last season in the 50-50 combination treatment, despite the ERY-DOX synergy (vertical bars are s.e., $n = 6$).

§3 Additional Data

3.1 The combination treatment eventually loses out to all sequential treatments at IC_{50}

The data presented in the figures of this section (Fig. S9, Fig. S10) demonstrate that a treatment producing the greatest inhibitory effect when measured over a single season need not continue to inhibit growth maximally as treatment proceeds. At these dosages, the 50-50 combination treatment is optimal at reducing population growth in season 1, but it is close in performance to the worst treatment of all by season 8.

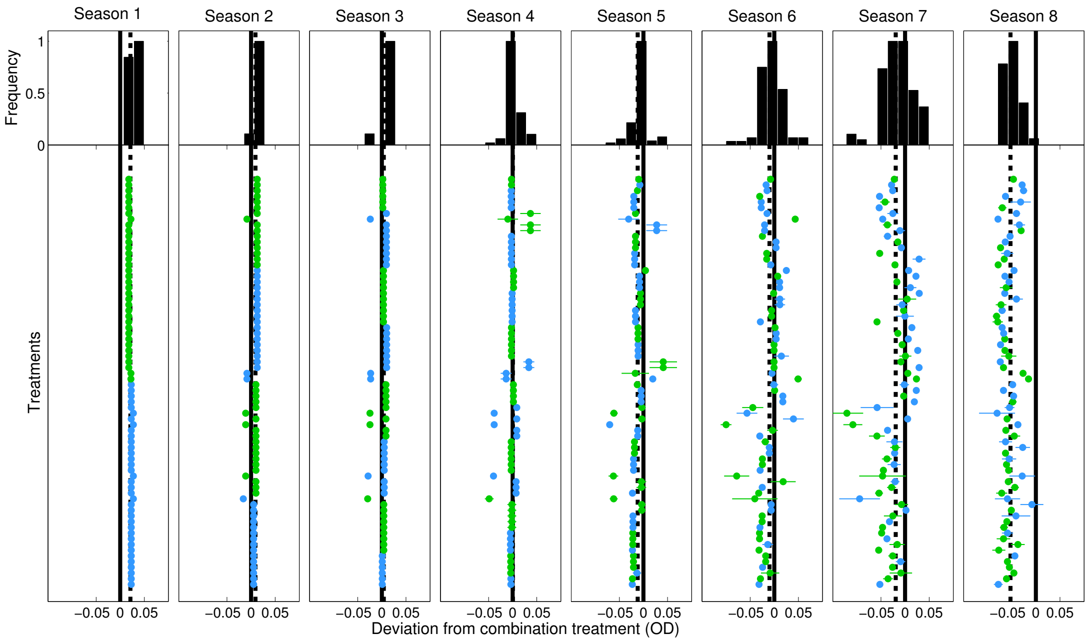

Fig. S9 – Deviation in OD from the combination treatment at the end of each season for all sequential treatments tested. Vertical thick black lines denote the mean OD of the 50-50 combination treatment, normalised and written as zero, vertical dotted lines represent the mean OD taken over all the sequential treatments and the coloured dots show the deviation of the OD of each sequential treatment from the 50-50 combination (blue dots: DOX, green dots: ERY). The histograms represent the distributions of performances of all sequential treatments relative to the combination treatment in each season. This series of so-called forest plots show how at the beginning of the experiment all dots are to the right-hand side of the 50-50 line: their performance is worse than the multidrug combination. However, as the number of seasons increases, more treatments cross the vertical line and thus achieve higher inhibition than the 50-50 combination. By the end of the experiment all sequential treatments outperform the multidrug combination.

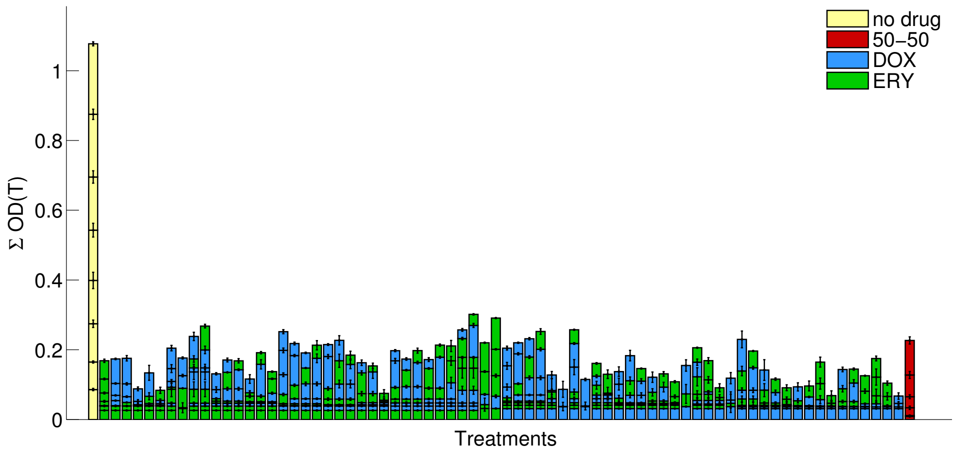

Fig. S10 – A 'Manhattan plot' at IC_{50} dosages (3 replicates per treatment): $\Sigma \mathsf{OD}(\mathsf{T})$ (on the y axis) denotes the cumulative optical densities measured at the end of each season and these values are summed to produce a 'skyscraper' for each treatment. Here T denotes 12h, the duration of each season, so $\mathsf{OD}(\mathsf{T})$ represents population density at the end of each season. Vertical error bars are s.e., $n = 3$.

3.2 No correlation between drug switching and inhibitory effect at IC_{50} and IC_{70} dosages

We first determined the ODs produced by all the balanced sequential treatments at IC_{70} dosages and, as shown in Fig. S11, we sought a negative correlation between the number of switches in an 8-season treatment and the number of bacteria it produces. This was done in two ways: we measured the total number of bacteria produced by each treatment (using OD as the proxy) and we measured the final OD produced, namely at 96h. We then compared the number of drug switches with these population density measures produced by each treatment. This figure shows no evidence of the correlation we sought.

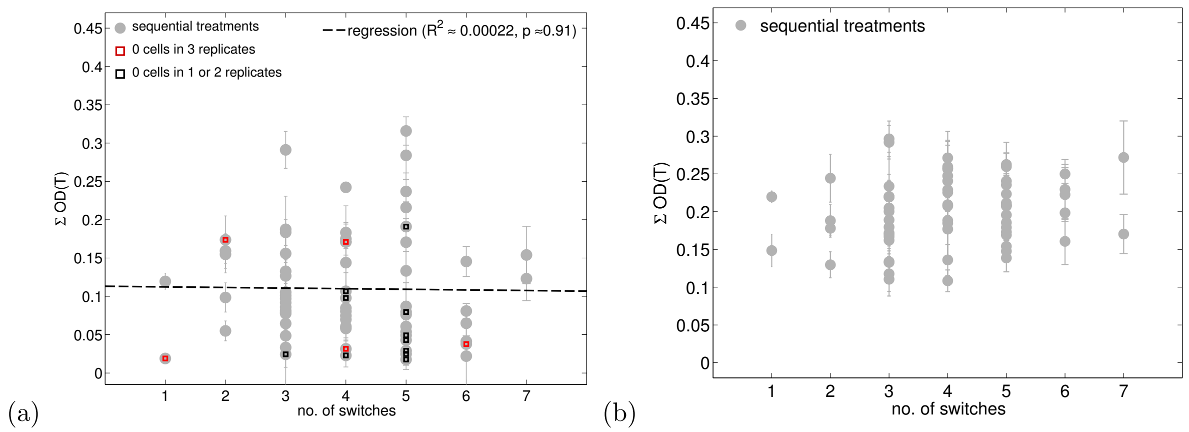

Fig. S11 – (a) At IC_{70} there is no correlation between the number of drug switches and the mean total number of bacteria produced per sequential treatment. Here the 16 best performing IC_{70} treatments, those with lowest population densities at 96h in Figure 2A of the main text, are highlighted with red and black squares (error bars are s.e., $n = 3$). The black line is a linear regression between the number of drug switches and the (cumulative) OD data, showing no evidence of a correlation. The data in (b) is analogous to (a) but it was produced at IC_{50} dosages.

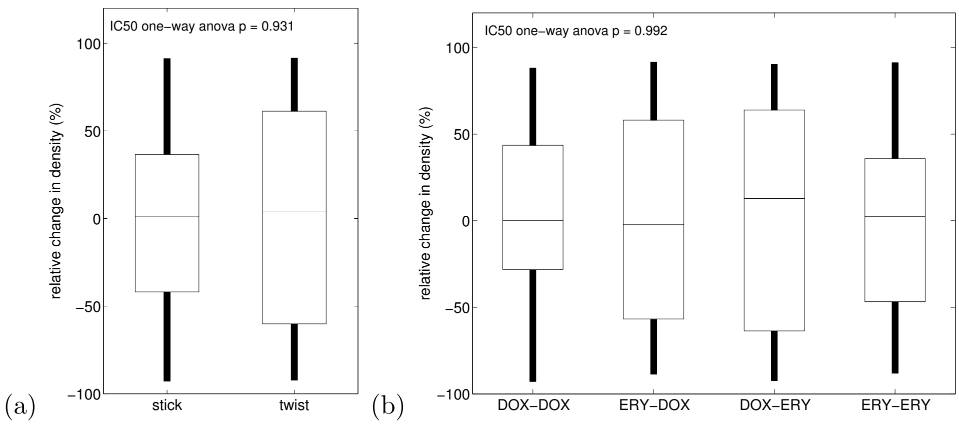

Fig. S12 – H_0: effecting a change of drug correlates with a measurable change in OD. (a) This boxplot of relative change in OD from one season to the next (namely $(y-x)/(x+y)$ where x is OD at 12h on day n and y is OD at 12h on day $n+1$, expressed as a percentage) provides no evidence to reject H_0 using the same dataset as used in Fig. S10. Here $(y-x)/(x+y)$ (that, note, necessarily lies between -1 and $+1$) is computed for two treatment classes: 'twist' treatments occur when ERY follows DOX or DOX follows ERY from day n to day $n+1$, 'stick' treatments occur when DOX follows DOX or ERY follows ERY. (b) Stratifying the same treatments further is not sufficient to reject H_0.

3.3 Some sequential treatments, but not the combination, clear the bacterium at IC_{70} dosages

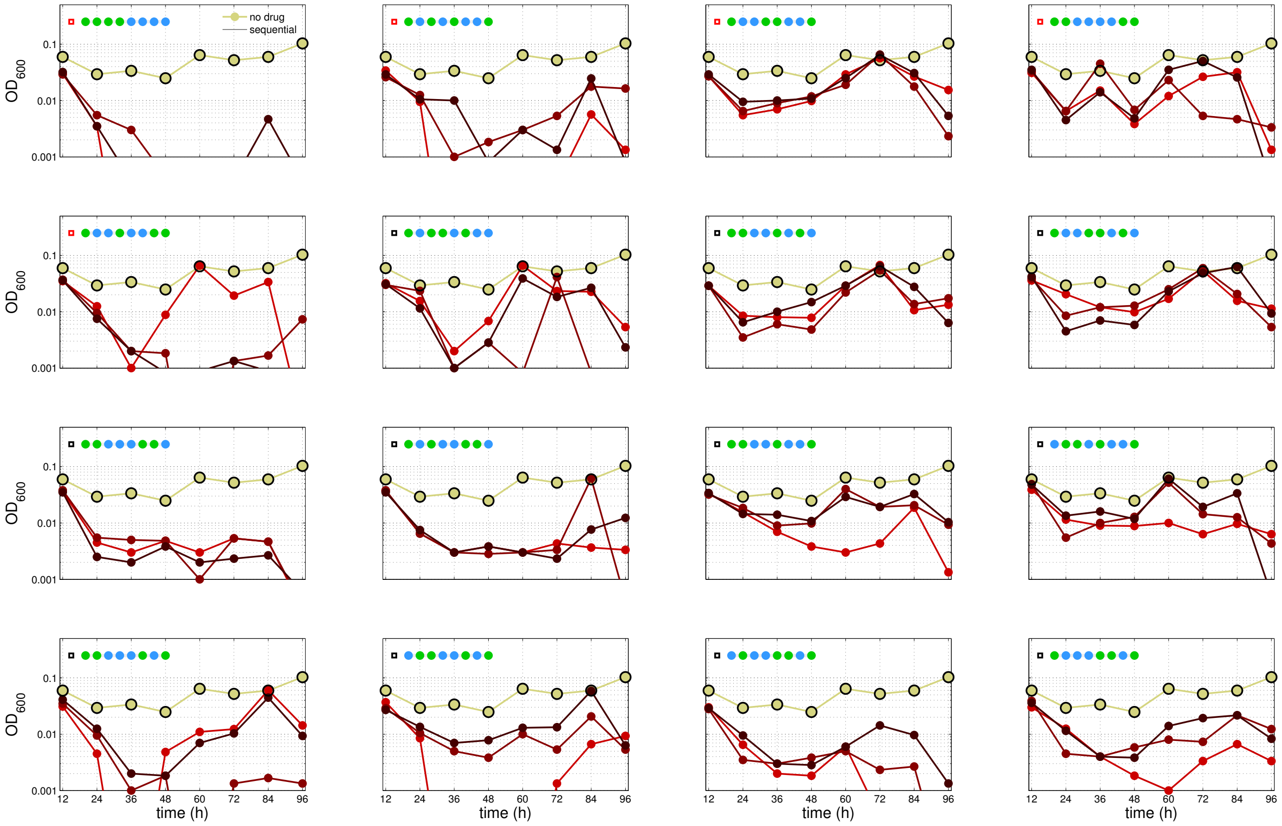

Fig. S13 – Sixteen different sequential ERY-DOX treatments were replicated at IC_{70} three times, each of these replicates is compared with the zero-drug control (the former in one of three shades of red, the latter in yellow). In the 5 of these having red squares in the legend, a spot test indicated no live cells in the growth medium after 96h of treatment. Of the remaining 11, in 4 there were no live cells found in 2 replicates and 7 treatments in which a single replicate had no live cells at 96h. Thus, a total of 48 sequential treatments were trialled at sub-lethal dosages, 30 of which lead to complete elimination of the bacterium by 96h. By way of contrast, and as expected, Fig. S14 shows that monotherapies and the 50-50 combination treatment do not eliminate bacteria at these dosages. The drugs used for each treatment are indicated in each panel, with a blue dot for DOX and a green dot for ERY.

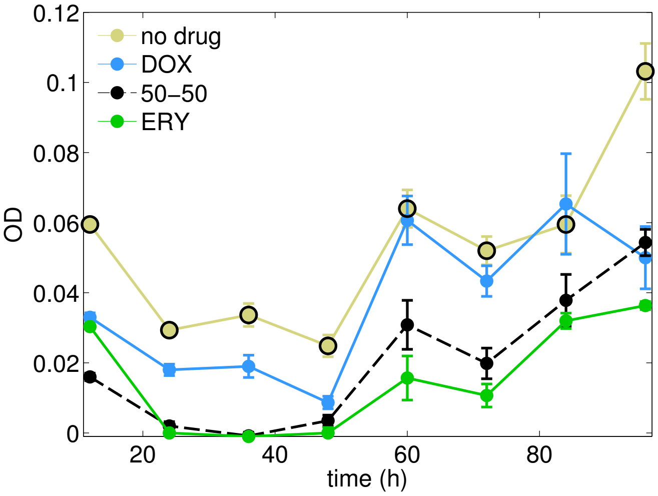

Fig. S14 – Monotherapies and 50-50 combination treatments at IC_{70} and the zero drug control, *c.f.* Fig. S13. This illustrates the short-term synergy, the loss of synergy, it also highlights that these treatments lead to increasing population densities at 96h (error bars are s.e. with $n = 3$).

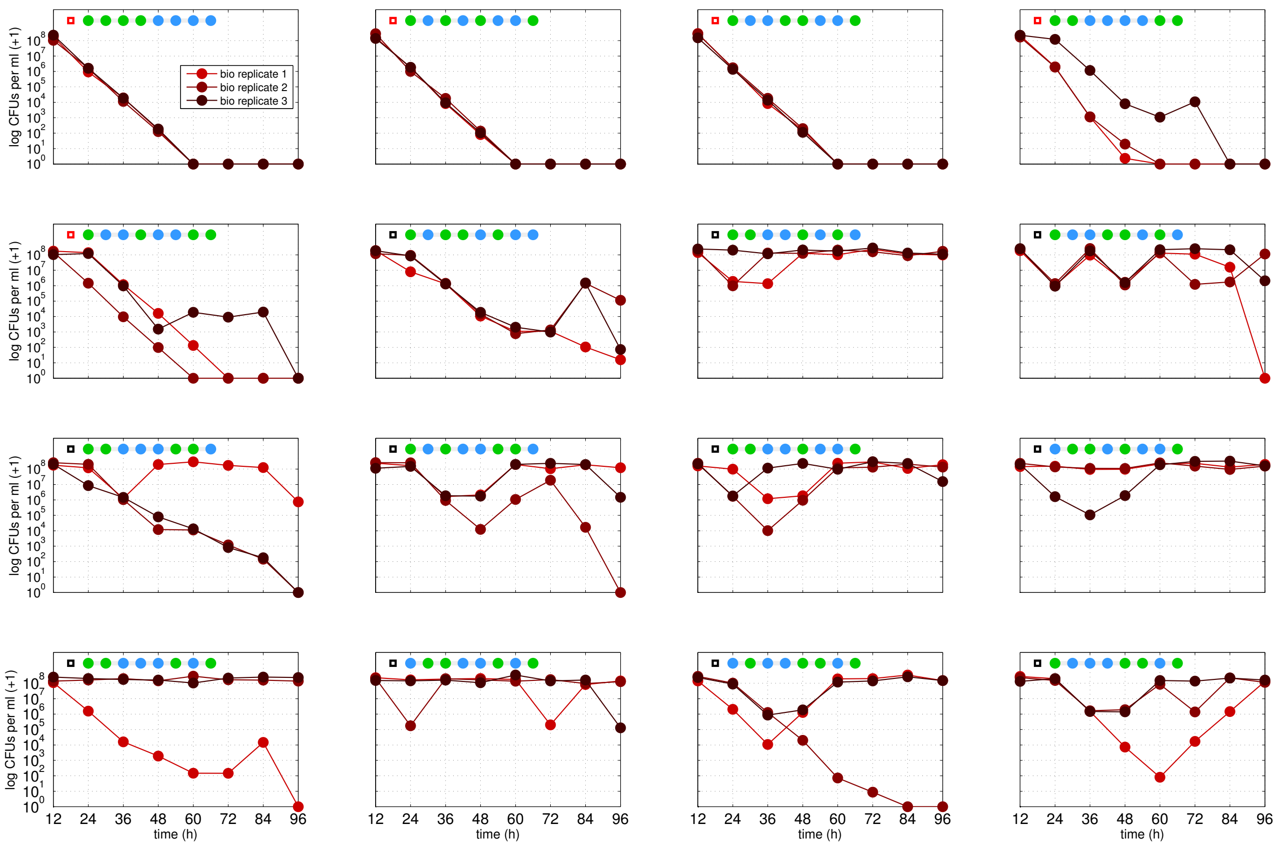

Fig. S15 – Dynamics of replicates of the sequential treatments at IC_{70} shown in Fig. S13 that indicated a zero population density in at least one replicate. These were replicated and cell densities counted each season, thus colony forming units (CFUs) are now used on the y-axis (in fact, $\log_{10}(1+C)$ is shown where C is cell density measured in CFUs per ml which is zero if $C = 0$.) The antibiotic treatments are indicated as a legend in each figure. Note how the population density of one replicate can eventually collapse for a given treatment whilst a different replicate can recover, leading to large between-replicate variations. Note also that this replicated dataset has 21 successful treatments from the full set of 48 replicates. This is a different outcome to the first set of replicates shown in Fig. S13, however the so-called 'red square' treatments behave consistently in all replicates by producing a zero CFU count at some time.

§4 A whole genome sequencing analysis

In order to investigate antibiotic resistance adaptation based on prior and *de novo* mechanisms, we performed the following analysis. We cultured bacteria with 12h seasons using a batch transfer protocol implemented in a shaken flask with 5ml of M9 liquid medium, implementing one environmental condition without drugs (called the 'no drug control') and two different antibiotic conditions. All were replicated three times. The two antibiotic treatments were the 50-50 combination at IC_{50} dosages and the sequential treatment of the from 'EDEDEDED'. Measuring a $150\mu L$ sample from the flask in a plate reader at 600nm, the cumulative optical densities produced were similar for both these drug treatments as shown in Fig. S16. All replicate populations from 24h and 96h were then sequenced using the paired end technology on a Illumina 7500 machine at the Exeter sequencing service. Both single-drug monotherapies and the 'DEDEDEDE' treatments were implemented as controls, but were not sequenced (see Fig. S16).

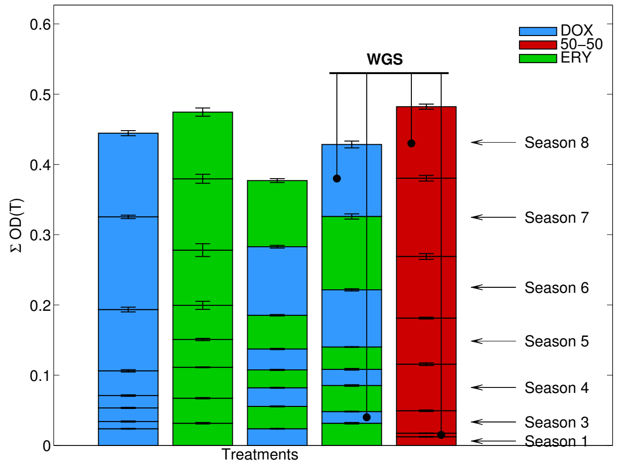

Fig. S16 – Cumulative optical density at the end of each season for five different treatments: two single-drug treatments (DOX in blue and ERY in green), a 50-50 multidrug combination (in red) and sequential treatment protocols based on a daily antibiotic switch. Note how the treatment with the highest cumulative optical density is the 50-50 combination, despite it being the most effective drug at the beginning of the experiment. The label WGS indicates the densities and timings of sequenced populations (3 replicates each).

4.1 Library preparation method

DNA was fragmented by sonication using a Biorupter for 30s on, 90s off, using low power for 10 minutes on ice. Libraries were prepared using SPRIworks cartridges for Illumina (Beckman Coulter) and Nextflex indexed adapters, with 300-600 bp size selection, amplified with 8 cycles PCR using Kapa HiFi DNA polymerase and purified using GeneRead kit (Qiagen). Concentrations were determined using a Bioanalyser 7500 DNA chip. Libraries were pooled in equimolar amounts, denatured, diluted to 6.5 pMol and clustered on a flowcell using a cBot (Illumina). 100 paired end sequencing with a custom barcode read was completed on a HiSeq 2500 using Truseq SBS v3 reagents (Illumina).

4.2 Construction of a Local Reference

To facilitate the genomic analysis we first constructed a local reference genome of *E. coli* K12 (AG100), constructed by modifying the publicly available annotated genome of strain MG1655[1] [19]. This choice was motivated by its close relation with AG100 and the quality of the existing annotation.

Reads were processed with fastq-mcf [2] to remove adapters from the sequencing data and to trim and filter low-quality reads. In particular, cycles with at least 1% of 'N's were removed (command-line parameter: `-x 0.01`). The remaining reads were mapped to MG1655 using the Burrows-Wheeler aligner BWA [13] with standard parameters. The resulting alignments were processed with Samtools 1.0 [14], with pair/trio calling enabled (command-line parameter: -T). Subsequently, alignments were sorted, artifacts and duplicates were removed, and finally the alignments was indexed. Unaligned reads were stored separately.

Structural Variations (SVs) were detected using Pindel [25]. Its pattern-growth algorithm detects breakpoints of large deletions and medium-sized insertions by identifying paired reads for which only one of the reads can be mapped to the reference. It then attempts to break the unmapped read into two and maps both shorter fragments to the reference. If successful, the breakpoints of deletions or insertions can thus be determined. We restricted the detection to SVs of maximum 2,071,552bp (command-line parameter: `-x 8`). The detected SVs were visually inspected with IGV [21, 23]. We validated 5 deleted regions, 9 putative breakpoints and 9 indels, nucleotide polymorphisms were intentionally ignored at this step.

[1]MG1655 genome and its annotation files are available at `ftp://ftp.ncbi.nih.gov/genomes/Bacteria/Escherichia_coli_K_12_substr__MG1655_uid57779/`; version NC_000913.2 of 22 July 20013 was used.

Using a Python script, we removed deleted regions and putative breakpoints detected by Pindel, resulting in a set of 15 non-overlapping sequences free of major SVs. Detected indels were subsequently applied to these sequences. With Samtools we extracted the reads previously mapped to removed regions or within 2/3 of the library insert size from their borders. These and the reads without a feasible alignment were assembled with Velvet [26] resulting in a set of contigs. The union of this set and the set of the 15 sequences was used to generate a set of scaffolds using SSPACE [3]. This software uses the distance information of paired-ends to assess the order, distance and orientation of the submitted contigs and combines them into scaffolds. The resulting scaffolds were extended with GAPFILLER [4] that attempts to close gaps between scaffolds using the distance information of paired-read data; 21,942 scaffolds were thus obtained.

These scaffolds were aligned to MG1655 using Mauve [20]. This software can align a set of sequences to one genome from a related one that differs from the former for the presence of SVs and only the 13 longest sequences (the shortest was 109,280 bp) could be aligned to the reference. The remaining sequences, not more than 2,375 bp each, were discarded. The 13 scaffolds were concatenated into one sequence, here called the 'intermediate' reference according to an order and orientation given by Mauve.

We used Pindel to verify if SVs were still present in the intermediate reference and to validate the multiple nucleotide polymorphisms detected in the previous execution of Pindel. No SV was observed visually with IGV, while 6 polymorphisms of 2 consecutive nucleotides each, detected even before by Pindel, were still present. We applied these polymorphisms to the intermediate reference using the script vcf-consensus of the vcf-tools utility [6]. Finally, SNPs were detected using the software VarScan.

VarScan uses both heuristic and statistical methods to call SNPs based on read depth, base quality, significance and variant frequency. We used VarScan with the following parameters: p-value threshold of 0.05 for calling variants, minimum read depth of 20 to make a call at a position, at least 8 supporting reads at a position to call variants, base quality not less than 20 at a position to count a read, minimum variant allele frequency of 0.03; frequency to call homozygote of at least 0.9 (command-line parameters: `--p-value 0.05 --min-coverage 20 --min-reads2 8 --min-avg-qual 20 --min-var-freq 0.03 --min-freq-for-hom 0.9`) The detected SNPs were applied to the intermediate reference with vcf-consensus and the resulting sequence was our local reference. The local reference was mostly annotated using RATT [17], transferring the annotation from MG1655 to this sequence for regions with high synteny. Using the RAST annotation server, we annotated the two longest regions with low synteny (37,193 bp and 7,166 bp, respectively).

4.3 Mapping onto local reference

All 18 replicates of three drug treatments were mapped to the local AG100 reference genome using the same method adopted for mapping the control replicate to the MG1655. As above, reads were processed with fastq-mcf and mapped to the local reference with BWA. Again, SNPs and SVs were called with VarScan and Pindel, respectively, using the same parameters previously adopted. Additionally, we used CNVnator [1] to discover copy number variations (CNVs). This software detects CNVs through an analysis of read mapping density (coverage) within different bins along the genome. A bin size of 60 was chosen for all CNVnator analyses.

4.4 SNP-detection heuristic

Let '0' denote a known wild-type allele at a given locus and suppose that Illumina sequencing produces n aligned reads covering this locus that is denoted by a sequence of alleles, $\{X_j\}_{j=1}^n$, where each X_j is a Bernoulli random value that only takes the values '0' or '1', the latter symbol (taking a numerical of unity) denoting any synonymous or non-synonymous mutation. Now define $Y_n = \frac{1}{n}\sum_{j=1}^n X_j$. Suppose that the systemic per-nucleotide error rate at each locus is ϵ, this is the probability that one Illumina read reports an incorrect allele following alignment.

A putative SNP acceptance rate of α per read (expressed as a value between 0 and 1) is here said to produce a false positive rate p that is defined to be the probability that n aligned reads reports at least $\alpha \times n$ occurrences of the allele '1' due to Illumina read error. This is the value $p := \mathbb{P}(nY_n > n\alpha) = \mathbb{P}(Y_n > \alpha)$. As each X_j can be modelled as a Bernoulli trial with parameter ϵ, for sufficiently large n the probability distribution of Y_n can be approximated by a normal distribution with the following mean and variance parameters (following standard notation) $\mu := \mathbb{E}(Y_n) = \epsilon$ and $\sigma^2 := \mathrm{var}(Y_n) = \epsilon(1-\epsilon)/n$. Thus

$$p = \mathbb{P}(Y_n > \alpha) \approx \frac{1}{\sigma\sqrt{2\pi}}\int_\alpha^\infty e^{-(x-\mu)^2/2\sigma^2}dx = \frac{1}{\sqrt{\pi}}\int_q^\infty e^{-x^2}dx = \frac{1}{2}\mathrm{erfc}(q),$$

following the change of variable, $q = (\alpha-\mu)/(\sigma\sqrt{2})$. Using

$$\mathrm{erfc}(q) \approx \frac{e^{-q^2}}{q\sqrt{\pi}}\left(1 - \frac{1}{2q^2} + O(q^{-4})\right),$$

we deduce the following approximation for the false positive SNP acceptance rate for given α:

$$p \approx \frac{e^{-n(\alpha-\epsilon)^2/2\epsilon(1-\epsilon)}}{(\alpha-\epsilon)}\left(\frac{2\epsilon(1-\epsilon)}{n\pi}\right)^{1/2}(1+O(n^{-1})),$$

where $\alpha > \epsilon$. Using a value $\epsilon = 10^{-2}$ [16, 15] and a mean observed value for coverage of $n \approx 200$ (this is representative of values given in Fig. S17), with this calculation, shows a putative variant frequency of $\alpha = 2\%$ yields the value $p \approx 0.0776$ whereas using $\alpha = 3\%$ yields $p \approx 2.24 \times 10^{-3}$. The requirement that $p < 0.05$ thus permits us only to report SNPs with an estimated frequency of 3% or greater.

4.5 WGS Results

Our analysis indicated a 412,380bp duplication (from 273,601 to 685,980) in all 6 drug treatment replicates at 96h. After a list of putative SNPs was first generated in the manner detailed above, Table S1,Table S3 and Table S4 then summarise only those SNPs for which significant longitudinal or between-treatment differences are observed using a one or two-way anova with a threshold of $p < 0.1$. Putative SNPs that do not pass this acceptance criterion have not been reported. Genes annotated using red font are contained within the duplicated region. For completeness, Table S5 contains basic statistics used in the whole-genome analysis.

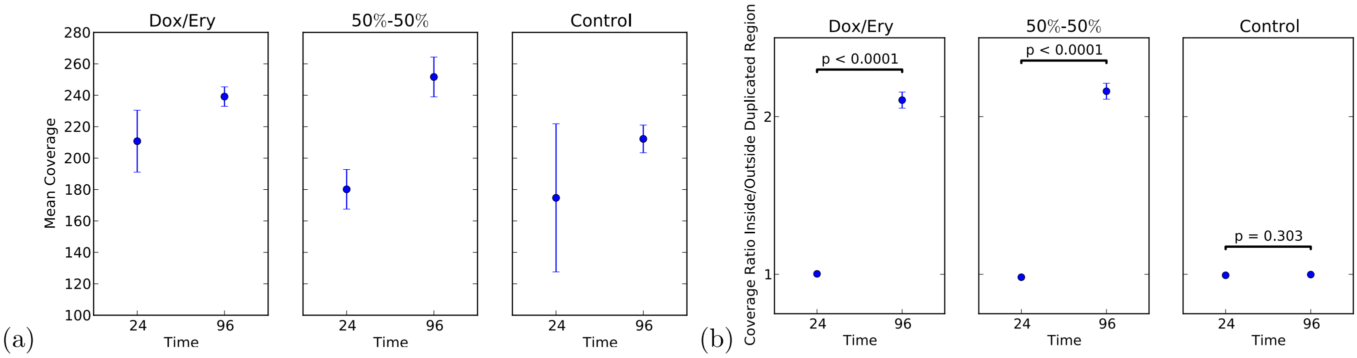

Fig. S17 – (a) Mean coverage (three replicates) of the different drug-treated and drug-free sequenced populations, ± s.e., $n = 3$. (b) Evidence for a duplicated genomic region given by the ratio of mean coverage in two contiguous regions: inside and outside addresses 273,601bp to 685,980bp. There is no evidence of the duplication (where this ratio equals 2) in the absence of drug treatment, but there is evidence that it is duplicated in all drug-treated populations by 96h. The indicates p-values are for t-tests seeking differences in mean coverage between 24h and 96h.

Comments regarding SNPs:

(1) The Ribosomal Mutation Database filtered by position for '595' (`http://goo.gl/sbFIFd`) only shows 16S (not 23S) SNPs in this location. The database indicates that none of the *rrn* SNPs in Table S1 are known tetracycline or erythromycin resistance mutations.

23S erythromycin resistance mutations in the database: `http://goo.gl/f7WrCH`

The database has a tetracycline resistance mutation but we do not observe it here: `http://goo.gl/0NsnJf`

(2) *atoB* is a short-chain fatty acid degradation enzyme (thiolase II) [11] in the *ato* operon.

(3) *tauA* is required for sulphur utilisation '*expressed only under conditions of sulfate or cysteine starvation*' [7]; cysteine is an α-amino acid in the casamino acids supplied at 0.1%.

(4) *mdtG* (*yceE*) is a putative efflux protein that confers resistance to *fosfomycin* and *deoxycholate*, also the quinolone *norfloxacin* (see `http://goo.gl/9pLxZ1`), it is a member of the *marA-soxS-rob* stress regulon that mediates expression of the *acrAB-tolC* pump [8].

(5) *yqhC* regulates *yqhD* where the latter is '*a scavenger of toxic aldehydes produced by lipid peroxidation*' [10] (see also '*we propose that YqhC is a transcriptional activator of YqhD, which acts as an aldehyde reductase*' [12]), as does *paoC* of Table S3.

(6) *ubiD* catalyses the synthesis of the antioxidant *ubiquinone* (`http://goo.gl/2Al4fS`) that '*can prevent or control chain lipid peroxidation in biomembranes*' [5].

(7) *slt* '*degrades the murein polymer of the bacterial cell wall to 1,6-anhydromuropeptides*' [22].

(8) *E.coli* prophage (including DLP12) can aid survival in '*adverse environments*' including osmotic, oxidative and acid stresses [24].

4.6 Tables of SNPs (Table S1, Table S3 and Table S4)

These summarise significant SNPs observed at 24h and 96h under the different treatment conditions. The mean frequency in the populations where the SNP was detected is indicated and a superscript denotes the number of replicates in which the SNP was observed. A blank entry denotes it was not detected.

Table S1 – Ribosomal SNPs: all belong to 23S.

			% mean variant frequency$^{\text{(replicates, if not all)}}$					
			**50%-50%**		**Dox/Ery**		**Control**	
operon	position	relative pos^{n}	24h	96h	24h	96h	24h	96h
rrnH	226,521	595						$5^{(2)}$
	227,791	1,865				$3^{(1)}$		17
rrnG	2,723,624	1,865				$3^{(1)}$		9
	2,724,894	595						8
rrnD	3,421,431	1,865				$4^{(1)}$		13
	3,422,701	595						8
rrnC	3,940,810	595				$4^{(1)}$		17
rrnA	4,034,586	555						7
rrnB	4,165,708	595				$4^{(1)}$		8
	4,166,978	1,865						10
rrnE	4,207,110	595				$3^{(1)}$		9
	4,208,380	1,865				$5^{(1)}$		7

Table S2 contains a list of genes within the duplicated genomic region that are known to interact with antibiotics in some way, whether through drug binding or efflux, or else are implicated in the regulation of stress pathways that may be associated with increased resistance.

Table S2 – Several antibiotic-binding and resistance genes are found in the duplicated genomic region, including their annotations. **note (1)**: *emrE* provides resistance against positively charged compounds including ethidium bromide and erythromycin; **note (2)**: *nfsB* reduces a broad range of nitroaromatic compounds, including antibiotics nitrofurazone and nitrofurantoin.

start pos	end pos.	gene	annotation
394,429 b.p.	395,586 b.p.	*ampH*	penicillin-binding protein
395,938	397,158	*sbmA*	peptide antibiotic transporter
444,601	445,965	*yajR*	YajR MFS transporter
451,369	452,844	*ampG*	muropeptide transporter
458,187	460,541	*lon*	DNA-binding ATP-dependent protease La
468,170	469,942	*mdlA*	predicted multidrug transporter subunit; ATP-binding component
469,935	471,716	*mdlB*	fused predicted multidrug transporter; ATP-binding components
480,553	483,702	*acrB*	multidrug efflux system
483,725	484,918	*acrA*	multidrug efflux system
485,060	485,707	*acrR*	regulates the *acrAB* operon
502,775	503,995	*fsr*	fosmidomycin efflux transporter
515,882	516,661	*ybbM*	putative transport; drug/analog sensitivity
567,614	567,946	*emrE*	member of the SMR family of transporters (see note (1))
592,541	593,914	*cusC*	copper/silver efflux system, outer membrane component
594,420	595,643	*cusB*	copper/silver efflux transport system - membrane fusion protein
595,655	598,798	*cusA*	copper/silver efflux system, membrane component
601,712	602,416	*nfsB*	oxygen-insensitive NAD(P)H nitroreductase (see note (2))
659,693	660,976	*dacA*	penicillin-binding protein
662,142	663,254	*mrdB*	rod shape-determining membrane protein
663,257	665,158	*mrdA*	penicillin-binding protein

Table S3 – SNPs located neither in prophage nor ribosomal genes.

		% mean variant frequency$^{\text{(replicates, if not all)}}$						
		**50%-50%**		**Dox/Ery**		**Control**		
gene	position	24h	96h	24h	96h	24h	96h	annotation
ampE	119,553 b.p.	$8^{(2)}$	$6^{(1)}$		$6^{(1)}$	$13^{(1)}$		regulator; ampicillin resistance inner membrane protein
								**duplicated region** (red text denotes duplicated genes)
paoC	298,353	$9^{(1)}$	$7^{(2)}$		$5^{(2)}$			aldehyde oxidoreductase, moco-containing subunit
tauA	384,897				$19^{(1)}$		68	taurine transport system periplasmic protein
ybbJ	513,924				$4^{(2)}$			inner membrane protein, stimulates mutant suppression
mdtG	1,157,546	21	24	25	19	$17^{(1)}$	24	multidrug resistance efflux transporter
atoB	2,329,327	$8^{(1)}$		$7^{(1)}$	8		$9^{(1)}$	acetyl-CoA acetyltransferase
yqhC	3,151,384						45	putative ARAC-type regulatory protein
rng	3,394,063						$6^{(2)}$	endoribonuclease involved in 16S rRNA processing
ubiD	4,022,772				$20^{(1)}$		$10^{(2)}$	3-octaprenyl-4-hydroxybenzoate decarboxylase
rhaM	4,089,608		$8^{(2)}$	$7^{(2)}$	$9^{(1)}$	$7^{(2)}$	$7^{(2)}$	L-rhamnose mutarotase
slt	4,628,265	$9^{(1)}$	10	$7^{(1)}$	9	$6^{(1)}$	11	soluble lytic murein transglycosylase

Table S4 – Prophage-related SNPs.

		% mean variant frequency$^{\text{(replicates, if not all)}}$						
		**50%-50%**		**Dox/Ery**		**Control**		
gene	position	24h	96h	24h	96h	24h	96h	annotation
ybcV	576,213 b.p.			$37^{(2)}$	$58^{(1)}$	$35^{(1)}$	$41^{(1)}$	DUF1398 family protein
ybcW	576,962	47	64	49	68	48	51	phage or prophage related, unclear function
ylcI	577,244	51	68	52	69	49	52	DUF3950 family protein
rzoD	577,639	9	3	$9^{(2)}$	$5^{(2)}$	$11^{(2)}$	7	DLP12 prophage protein
rzpD	577,772	14		$20^{(2)}$	$12^{(1)}$	17	17	over-expression causes abnormal biofilm architecture (prophage DLP12)
nohD	577,984	54	37	49	36	50	55	DNA packaging (DLP12 prophage protein)
nohQ	1,639,729	14	8	12	9	13	10	DNA packaging
ydfJ	1,640,037	49	46	45	42	45	42	prophage related, putative transport protein
insH1	3,126,511		$7^{(1)}$		$7^{(1)}$	$10^{(1)}$	$9^{(1)}$	transposon related

Table S5 – Basic statistics for Illumina reads: statistics of the reads averaged over 3 replicates.

(a)

**DOX/ERY sequential**	mean (st. error)	
	24h	96h
Number of reads	13,289k (1,161k)	15,028k (429k)
Read max length	99.0 (0.0)	99.0 (0.0)
Read mean length	74.2 (0.8)	74.2 (0.8)
Read min length	15.0 (0.0)	15.0 (0.0)
Mapped reads (%)	98.8 (0.2)	99.3 (0.1)
Mean Mapping Quality	58.8 (0.01)	58.8 (0.0)

(b)

**50-50% combination**	mean (st. error)	
	24h	96h
Number of reads	11,420k (669k)	15,831k (821k)
Read max length	99.0 (0.0)	99.0 (0.0)
Read mean length	73.3 (0.8)	74.3 (0.8)
Read min length	15.0 (0.0)	15.0 (0.0)
Mapped reads (%)	99.5 (0.03)	99.2 (0.1)
Mean Mapping Quality	58.8 (0.01)	58.8 (0.0)

(c)

**No-drug control**	mean (st. error)	
	24h	96h
Number of reads	11,691k (3,091k)	13,249k (552k)
Read max length	99.0 (0.0)	99.3 (0.3)
Read mean length	71.1 (0.9)	75.3 (0.07)
Read min length	15.0 (0.0)	15.0 (0.0)
Mapped reads (%)	96.6 (0.4)	98.5 (0.04)
Mean Mapping Quality	58.8 (0.01)	58.8 (0.0)

§5 An Additional Simulation of the Mathematical Model

The mathematical model featured in the main text and implemented using the parameter values in Table S6 shows that an asymmetric collateral sensitivity between ERY and DOX can arise for the following reason. The efflux pump is predicted to have different affinities for ERY and DOX, this causes different rates of selection for cells that express the pump and, also, for cells that duplicate the pump. This means different population structures are supported by the use of either drug in monotherapy.

To show this can be the basis of an ACS using the model, we first choose antibiotic supply concentrations for both drugs at their IC_{50} (measured at 24h) in the model (main text, third column in Fig. 5A). This same figure from the main text then shows that the population of bacteria adapts during five days of treatment with ERY, with increases in the number of cells both expressing the efflux pump and having duplications of the pump genes.

In the mutation-selection equilibrium indicated by this figure, the population converges to a stable configuration with population densities below ones that are achieved under no drug conditions. However, a switch to DOX in the model sees a change in the relative frequency of pump duplications but, for instance, because the pump in the model has different efficiencies at removing the two drugs from within the cell, this exchange in drug sees population densities rapidly increase. In the figure, after the switch the population soon achieves densities close to those observed without the drug. The third column, bottom plot of Fig. 5A and then Fig. 5B show analogous, but opposite, effects when the drugs are exchanged in the opposite sense.

Thus there are a number of factors that can contribute to an asymmetric collateral sensitivity: different efficiencies of the pumps at effluxing the drugs, the different affinities of the drug for their targets, the different reductions in absolute fitness attributable to each drug, the mutation rates associated with the duplications and the selection for each of those mutations resulting from these factors that control the rate of sweep of the duplications. Given such physical and evolutionary asymmetries, the model exhibits both a cross resistance and a collateral sensitivity, as the terms are defined in the text.

Fig. S18 shows that the population densities produced by each treatment depends, even in this theoretical model, on the way in which drugs are sequenced. Although both drugs are calibrated in the figure to an IC_{50} dose with respect to a population consisting almost of entirely of wild-type cells that do not carry duplications of the pump, as treatments progress the population structures do change. This can mean, as in Fig. S18, that some sequential treatments produce more bacteria than the 50-50 combination, whereas some produce fewer. Indeed, of the treatments shown in Fig. S18, it is a sequential treatment that produces fewest cells of all.

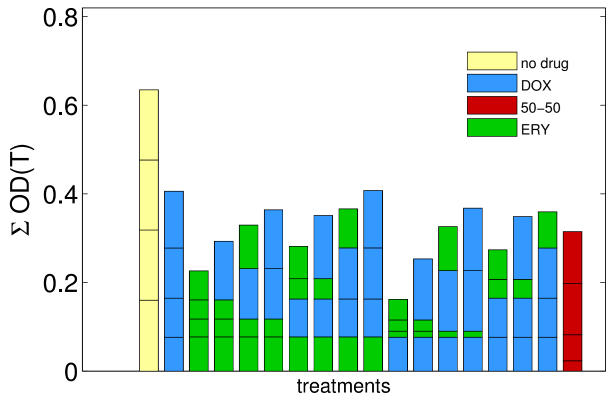

Fig. S18 – A Manhattan plot computed using the theoretical model from the main text as determined at IC_{50} dosages. Note how the sequential treatments (here of four seasons' duration) can be either better, or worse, than the 50-50 combination treatment depending on the orders in which the drugs are switched. Note the sequential treatment that produces fewest cells of all is the unbalanced 'DEEE' treatment.

Remark 1. *The parameter values given in Table S6 give rise to optical densities in the mathematical model (at* 0.2% *glucose) that are substantially larger than those reported in the experimental data throughout this article. This is not an error, rather the stated parameters were determined by calibrating the mathematical model in the main text against optical density data read at 600nm in 384 well plates* [18]. *With similar liquid volumes as the 96 well plates we use here, the path length taken for the absorbance measurement is necessarily larger and, therefore, similar population densities will result in very different optical densities between that article and this. Therefore, an optical density conversion factor of approximately* $\frac{1}{4}$ *OD units is required to compare data from this article and the mathematical model defined in* [18] *that we use here.*

Table S6 – All parameters used in the mathematical model are defined in [18, Supplementary Information]. Units are defined as follows: $[S_0] = [E_0] = [D_0] = \mu g/ml, [c]$ =OD/$\mu g, [V] = \mu g$/OD/$h, [K] = \mu g/ml, [\kappa_e] = [\kappa_d] = ml/\mu g, [\kappa_{ed}] = (ml/\mu g)^2, [v_e] = [v_d] = ml$/OD/$h, [k_e] = [k_d]$ = dimensionless, $[\varphi_e] = [\varphi_d] = ml$/OD/$h, [\delta]$ = per genome/h, $[\Delta]$ = dimensionless = $[g], [d_D] = [d_E]$ = per day.

**Description**	**Parameters**	**Value** (source [18])
glucose supply at 0.2%	S_0	2,000
antibiotic supply (model IC_{50} @ 24h) for (ERY,DOX)	(E_0, D_0)	(13,0.138)
growth kinetics	(c, V, K)	(0.000315,1140,0.54)
inhibitory responses to antibiotics	$(\kappa_e, \kappa_d, \kappa_{ed})$	(0.2, 300, 4,000)
antibiotic uptake and efflux kinetics (ERY)	(v_e, k_e, ϕ_e)	(4000, 19.7, 93.1)
antibiotic uptake and efflux kinetics (DOX)	(v_d, k_d, ϕ_d)	(4000,0.8,0.041)
duplication rate, Poisson loss rate	(δ, Δ)	$(10^{-4}$,18)
drug decay parameters	d_D, d_E	in the interval $[\frac{1}{24}\ln(0.8), 0]$
diminishing rate of returns of new pumps per duplication	g	0.5

References

[1] A. Abyzov, A. E. Urban, M. Snyder, and M. Gerstein, *Cnvnator: An approach to discover, genotype, and characterize typical and atypical cnvs from family and population genomic sequencing*, Genome Research **21** (2011), 974–984.

[2] Erik Aronesty, *Comparison of sequencing utility programs*, The Open Bioinformatics Journal (2013), no. 7, 1–8.

[3] Marten Boetzer, Christiaan V. Henkel, Hans J. Jansen, Derek Butler, and Walter Pirovano, *Scaffolding pre-assembled contigs using SSPACE*, Bioinformatics **27** (2011), no. 4, 578–579.

[4] Marten Boetzer and Walter Pirovano, *Toward almost closed genomes with GapFiller*, Genome Biology **13** (2012), no. 6, R56.

[5] L Cabrini, P Pasquali, B Tadolini, A M Sechi, and L Landi, *Antioxidant behaviour of ubiquinone and beta-carotene incorporated in model membranes.*, Free Radic Res Commun **2** (1986), no. 1-2, 85–92 (eng).

[6] Petr Danecek, Adam Auton, Goncalo Abecasis, Cornelis A. Albers, Eric Banks, Mark A. DePristo, Robert E. Handsaker, Gerton Lunter, Gabor T. Marth, Stephen T. Sherry, Gilean McVean, Richard Durbin, and 1000 Genomes Project Analysis Group, *The variant call format and VCFtools*, Bioinformatics **27** (2011), no. 15, 2156–2158.

[7] E Eichhorn, J R van der Ploeg, and T Leisinger, *Deletion analysis of the Escherichia coli taurine and alkanesulfonate transport systems.*, J Bacteriol **182** (2000), no. 10, 2687–2695 (eng).

[8] Anna Fabrega, Robert G Martin, Judah L Rosner, M Mar Tavio, and Jordi Vila, *Constitutive SoxS expression in a fluoroquinolone-resistant strain with a truncated SoxR protein and identification of a new member of the marA-soxS-rob regulon, mdtG.*, Antimicrob Agents Chemother **54** (2010), no. 3, 1218–1225 (eng).

[9] Matthew Hegreness, Noam Shoresh, Doris Damian, Daniel Hartl, and Roy Kishony, *Accelerated evolution of resistance in multidrug environments.*, PNAS **105** (2008), no. 37, 13977–13981 (eng).

[10] Laura R Jarboe, *YqhD: a broad-substrate range aldehyde reductase with various applications in production of biorenewable fuels and chemicals.*, Appl Microbiol Biotechnol **89** (2011), no. 2, 249–257 (eng).

[11] L S Jenkins and W D Nunn, *Genetic and molecular characterization of the genes involved in short-chain fatty acid degradation in Escherichia coli: the ato system.*, J Bacteriol **169** (1987), no. 1, 42–52 (eng).

[12] Changhan Lee, Insook Kim, Junghoon Lee, Kang-Lok Lee, Bumchan Min, and Chankyu Park, *Transcriptional activation of the aldehyde reductase yqhd by yqhc and its implication in glyoxal metabolism of escherichia coli k-12.*, J Bacteriol **192** (2010), no. 16, 4205–4214 (eng).

[13] H. Li and R. Durbin, *Fast and accurate short read alignment with Burrows-Wheeler transform.*, Bioinformatics **24** (2009), 1754–1760.

[14] Heng Li, Bob Handsaker, Alec Wysoker, Tim Fennell, Jue Ruan, Nils Homer, Gabor Marth, Goncalo Abecasis, Richard Durbin, and 1000 Genome Project Data Processing Subgroup, *The sequence alignment/map format and samtools*, Bioinformatics **25** (2009), no. 16, 2078–2079.

[15] Nicholas J Loman, Raju V Misra, Timothy J Dallman, Chrystala Constantinidou, Saheer E Gharbia, John Wain, and Mark J Pallen, *Performance comparison of benchtop high-throughput sequencing platforms*, Nat Biotech **30** (2012), no. 5, 434–439.

[16] Dianne I. Lou, Jeffrey A. Hussmann, Ross M. McBee, Ashley Acevedo, Raul Andino, William H. Press, and Sara L. Sawyer, *High-throughput dna sequencing errors are reduced by orders of magnitude using circle sequencing*, Proceedings of the National Academy of Sciences (2013).

[17] Thomas D. Otto, Gary P. Dillon, Wim S. Degrave, and Matthew Berriman, *RATT: Rapid Annotation Transfer Tool*, Nucleic Acids Research **39** (2011), no. 9, e57.

[18] Rafael Pena-Miller, David Laehnemann, Gunther Jansen, Ayari Fuentes-Hernandez, Philip Rosenstiel, Hinrich Schulenburg, and Robert E Beardmore, *When the most potent combination of antibiotics selects for the greatest bacterial load: the smile-frown transition.*, PLoS Biol **11** (2013), no. 4, e1001540.

[19] Monica Riley, Takashi Abe, Martha B. Arnaud, Mary K.B. Berlyn, Frederick R. Blattner, Roy R. Chaudhuri, Jeremy D. Glasner, Takashi Horiuchi, Ingrid M. Keseler, Takehide Kosuge, Hirotada Mori, Nicole T. Perna, Guy Plunkett, Kenneth E. Rudd, Margrethe H. Serres, Gavin H. Thomas, Nicholas R. Thomson, David Wishart, and Barry L. Wanner, *Escherichia coli K-12: a cooperatively developed annotation snapshot-2005*, Nucleic Acids Research **34** (2006), no. 1, 1–9.

[20] Anna I. Rissman, Bob Mau, Bryan S. Biehl, Aaron E. Darling, Jeremy D. Glasner, and Nicole T. Perna, *Reordering contigs of draft genomes using the Mauve Aligner*, Bioinformatics **25** (2009), no. 16, 2071–2073.

[21] James T Robinson, Helga Thorvaldsdóttir, Wendy Winckler, Mitchell Guttman, Eric S Lander, Gad Getz, and Jill P Mesirov, *Integrative genomics viewer*, Nature Biotechnology **29** (2011), no. 1, 24–26.

[22] H J Rozeboom, B W Dijkstra, H Engel, and W Keck, *Crystallization of the soluble lytic transglycosylase from escherichia coli k12.*, J Mol Biol **212** (1990), no. 4, 557–559 (eng).

[23] Helga Thorvaldsdóttir, James T. Robinson, and Jill P. Mesirov, *Integrative Genomics Viewer (IGV): high-performance genomics data visualization and exploration*, Briefings in Bioinformatics **14** (2013), no. 2, 178–192.

[24] Xiaoxue Wang, Younghoon Kim, Qun Ma, Seok Hoon Hong, Karina Pokusaeva, Joseph M. Sturino, and Thomas K. Wood, *Cryptic prophages help bacteria cope with adverse environments*, Nat Commun **1** (2010), 147.

[25] K. Ye, M.H. Schulz, Q. Long, R. Apweiler, and Z. Ning, *Pindel: a pattern growth approach to detect break points of large deletions and medium sized insertions from paired-end short reads*, Bioinformatics **25** (2009), no. 21, 2865–2871.

[26] Daniel R. Zerbino and Ewan Birney, *Velvet: Algorithms for de novo short read assembly using de bruijn graphs*, Genome Research **18** (2008), no. 5, 821–829.
